# Synthesis of thiophene-fused heptalenes by cycloaddition of azulenothiophenes with dimethyl acetylenedicarboxylate

**DOI:** 10.1038/s41598-020-69425-w

**Published:** 2020-07-27

**Authors:** Taku Shoji, Kota Miura, Yukino Ariga, Akari Yamazaki, Shunji Ito, Masafumi Yasunami

**Affiliations:** 1 Department of Material Science, Graduate School of Science and Technology, Shinshu University, Matsumoto, Nagano 390-8621 Japan; 2 Graduate School of Science and Technology, Hirosaki University, Hirosaki, Aomori 036-8561 Japan; 3 Department of Chemical Biology and Applied Chemistry, College of Engineering, Nihon University, Koriyama, Fukushima 963-8642 Japan

**Keywords:** Chemistry, Organic chemistry, Synthetic chemistry methodology

## Abstract

Heptalene has a fused structure of two cycloheptatrienes which is one of the non-aromatic bicyclic molecules with a 12π-electronic structure. We report herein the synthesis of thiophene-fused heptalene derivatives from the corresponding azulenothiophenes via cycloaddition reaction with dimethyl acetylenedicarboxylate. Their structure was clarified by single-crystal X-ray structural analysis. The electronic properties of the thiophene-fused heptalenes obtained by this study were characterized by UV/Vis and fluorescence spectroscopy measurements. The electrochemical features of these derivatives were also examined by voltammetry and spectroelectrochemical experiments.

## Introduction

**Figure 1:**
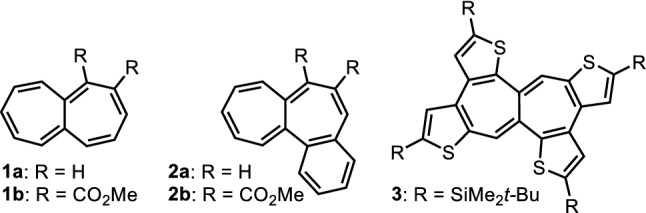
Heptalene derivatives **1a**, **1b**, **2a**, **2b**, and **3** appeared in the literature.

Heptalene has a fused structure of two cycloheptatrienes which is one of the non-aromatic bicyclic molecules with a 12π-electronic structure. Heptalene produces the stabilized aromatic dication and dianion with 10π- and 14π-electrons by two-electron oxidation and reduction, respectively. Fascinated by such unique properties, many researchers have studied the synthesis and characterization of various heptalene derivatives. Therefore, a number of synthetic methods for the heptalene derivatives have appeared in the literature. In 1961, the first synthesis of parent heptalene **1a** was reported by Dauben Jr. and Bertelli as an instable compound^1^. Hansen et al. established the preparation of benzo-fused heptalene **2a**, but **2a** is still unstable and the synthetic protocol requires a multi-step pathway^2^. Recently, Fukazawa, Yamaguchi, and their colleagues have achieved the synthesis of kinetically stabilized heptalene **3** fused by four thiophenes by reductive cyclization of bisdehydro[12]annulene with sodium metal, as well as its dianion (Fig. 1)^3^.

In addition to the above synthetic procedures, preparation of the heptalene skeleton is established by using the reaction of azulene derivatives with dimethyl acetylenedicarboxylate (DMAD). In 1976, Hafner et al. reported the cycloaddition reaction of azulene itself with DMAD to afford heptalene derivative **1b** with two-ester functions^4^. The reaction of benzo[*a*]azulene with DMAD gives benzene-fused heptalene derivative **2b**, which is demonstrated by Yasunami et al.^5^. Hansen et al. have reported the synthesis and reactivity of several fused-heptalenes including benzene-fused derivatives^6–15^. However, there are few examples of the synthesis of heptalenes by the reaction of ring-fused azulene derivatives with DMAD, and the properties of the ring-fused heptalene derivatives have hardly been evaluated, although a number of azulene derivatives may become a promising precursor for the heptalene synthesis.

In recent years, we have investigated the novel synthetic methods for various azulene derivatives and explored their distinctive reactivity^16–25^. In these researches, we have reported the synthesis, optical and electrochemical properties of azulenothiophene derivatives^23,26–28^ that become good precursors for the thiophene-fused heptalenes. Although thiophene-fused heptalene derivative **3** has already been reported as described above, preparation of much simpler derivatives should be essential for better understanding the nature of these series.

In this paper, we describe a synthesis of thiophene-fused heptalene derivatives from the readily accessible starting materials, i.e., azulenothiophenes. The structural feature of the heptalene derivatives was elucidated by single-crystal X-ray structural analysis. The electronic properties of the thiophene-fused heptalenes obtained by this study were characterized by UV/Vis and fluorescence spectroscopy and theoretical calculations. The electrochemical features of these derivatives were also examined by voltammetry and spectroelectrochemical experiments.

## Results and discussion

**Figure 2:**
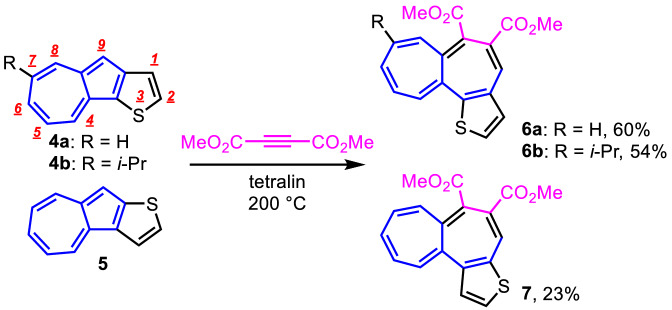
Synthesis of thiophene-fused heptalenes **6a**, **6b**, and **7** by the cycloaddition reaction of azulenothiophenes **4a**, **4b**, and **5** with DMAD; red-numbers show the numbering of ring-carbon.

Azulenothiophenes **4a**, **4b**, and **5**, precursors for the thiophene-fused heptalenes **6a**, **6b**, and **7**, were prepared by the reaction of 2*H*-cyclohepta[*b*]furan-2-ones with the corresponding enamines^29^. The reaction of azulenothiophene **4a** with DMAD in tetraline at 150 °C, subsequent chromatographic purification afforded the thiophene-fused heptalene **6a**, but in low yield (15%), whereas the reaction under the higher temperature condition (200 °C) resulted in a significant increase of the yield of **6a** (60%). Synthesis of **6b** and **7** was also accomplished by a similar manner by the reaction of **4b** and **5** in 54% and 23% yields, respectively (Fig. 2). Despite the parent heptalene **1a** is thermally unstable, compounds **6a**, **6b**, and **7** did not exhibit the decomposition even after standing for more than a year under the ambient conditions.

**Figure 3:**
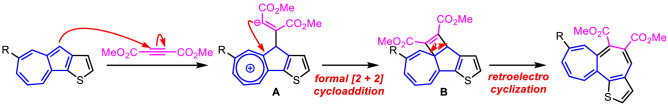
Presumed reaction mechanism for the formation of the thiophene-fused heptalenes.

The presumed reaction mechanism for the formation of the thiophene-fused heptalenes is illustrated in Fig. 3. The reaction should be initiated by the nucleophilic addition of electron-rich azulene at the 8-position to DMAD to produce the transient zwitterion **A**, which immediately cyclizes by intramolecular nucleophilic addition to give cyclobutene intermediate **B**. Eventually, strained cyclobutene intermediate **B** is transformed by the retroelectrocyclization to give the thiophene-fused heptalenes.

Thiophene-fused heptalenes **6a**, **6b**, and **7** obtained by this study were characterized on the basis of their spectral data as summarized in the “Methods” section. High-resolution mass spectra (HRMS) of the new compounds ionized by MALDI-TOF showed the correct molecular ion peaks. The characteristic stretching absorption of the carbonyl group was observed at ν_max_ = 1699 − 1701 cm^−1^ in their IR spectra, which supported the presence of the ester functions in these compounds.

**Figure 4:**
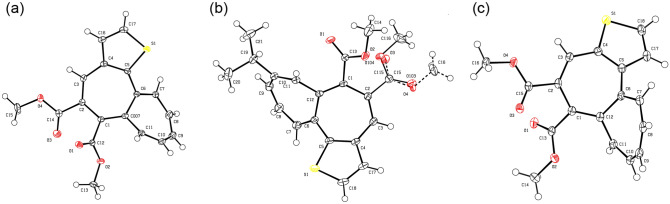
ORTEP drawings of thiophene-fused heptalenes (**a**) **6a** (CCDC1965949), (**b**) **6b** (CCDC1965950), and (**c**) **7** (CCDC1965951); ellipsoids are drawn at the 50% probability level.

**Figure 5:**
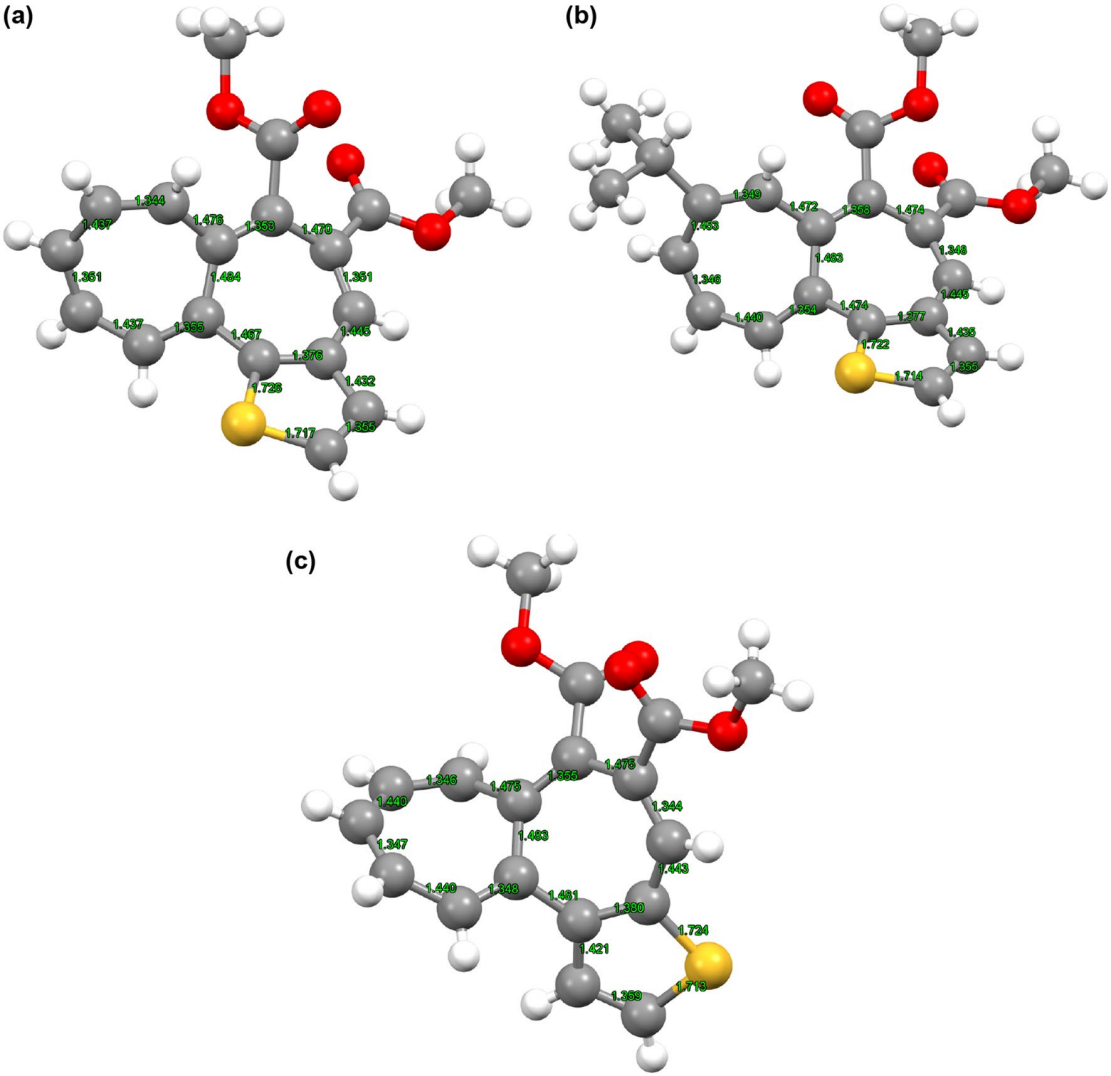
Bond length of heptalene moieties of (**a**) **6a**, (**b**) **6b**, and (**c**) **7**. The bond lengths in the single-crystal X-ray structure analysis were calculated using the following program; Mercury 4.1.0. Copyright CCDC 2001–2019 (https://www.ccdc.cam.ac.uk/mercury/).

Since the single crystals of **6a**, **6b**, and **7** were obtained by the slow evaporation of CH_2_Cl_2_/MeOH mixed solvent, the molecular structure of **6a**, **6b**, and **7** was clarified by single-crystal X-ray structure analysis (Fig. 4). The X-ray analysis of **6a**, **6b**, and **7** revealed their twisted structure between the 2 and 7 membered rings. The dihedral angles between the mean planes of these rings are observed as 49.78° (**6a**), 49.29° (**6b**), and 55.02° (**7**), whereas higher planarity between the thiophene and cycloheptatriene rings was observed with the dihedral angles of 19.25° (**6a**), 20.77° (**6b**), and 24.18° (**7**). Overall, **7** showed the lowest flatness compared to those of **6a** and **6b**; this may suggest a steric repulsion between the ring protons on the thiophene and heptalene rings in **7**. Furthermore, both X-ray and ^1^H NMR spectral analyses denoted the bond alternation in the fused-ring structure as shown in Fig. 5 that means the non-aromatic character in the heptalene moiety of **6a**, **6b**, and **7**.

**Figure 6:**
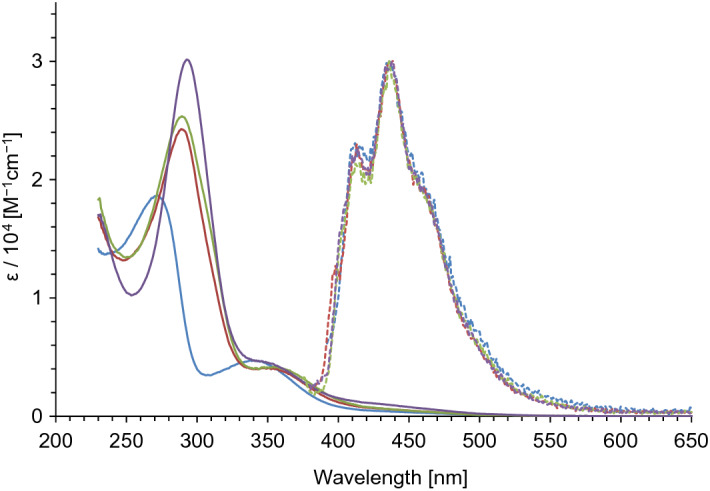
UV/Vis and fluorescence spectra of **1b** (blue line), **6a** (red line), **6b** (light-green line), and **7** (purple line) in CH_2_Cl_2_.

UV/Vis and fluorescence spectra of **6a**, **6b**, **7**, and **1b** in CH_2_Cl_2_ are shown in Fig. 6. The longest wavelength absorption maxima and their coefficients (log ε) are summarized in Table 1. The absorption maxima of **6a**, **6b**, and **7** exhibited a bathochromic shift by 10 − 13 nm compared with that of **1b**. In comparison with the longest wavelength absorption maximum of **3** (λ_max_ = 399 nm), that of **6a**, **6b**, and **7** showed hypsochromic shift by 45 − 48 nm, although no significant differences among those of **6a**, **6b**, and **7** were observed. These results indicate that the fused-thiophene ring contributes to the expansion of the conjugated system, but this arrangement does not have a significant effect with respect to their UV/Vis spectra. Thiophene-fused heptalenes **6a**, **6b**, and **7** in CH_2_Cl_2_ showed weak luminescence at λ_flu_ = 436 − 439 nm. Since heptalene **1b** also displayed the weak fluorescence in the similar region (λ_flu_ = 436 nm), this emission should originate from the heptalene skeleton.

**Table 1:** Absorption (λ_max_) and fluorescence maxima (λ_flu_) of heptalenes **6a**, **6b**, **7**, and **1b** in CH_2_Cl_2_.

Sample	λ_max_ (log ε)	λ_flu_	Stokes shift [cm^−1^]
6a	351 (3.61)	439	5.71 × 10^3^
6b	354 (3.62)	436	5.31 × 10^3^
7	354 (3.65)	438	5.42 × 10^3^
1b	341 (3.67)	436	6.39 × 10^3^

As mentioned in the Introduction section, heptalene derivatives generate the aromatic dication and dianion by two-electron oxidation and reduction, respectively. Heptalenes **6a**, **6b**, and **7** may produce the stabilized dication and dianion by the electrochemical redox reaction. Thus, the redox behavior of **6a**, **6b**, and **7** was examined by cyclic voltammetry (CV) and differential pulse voltammetry (DPV) experiments (Table 2). The cyclic voltammograms of **6a**, **6b**, and **7** are summarized in the Supporting Information. Contrary to our expectations, heptalenes **6a**, **6b**, and **7** exhibited irreversible oxidation and reduction waves on the CV. Irreversibility on the CV waves of these compounds should be attributed to the instability of the cationic and anionic species generated by the electrochemical oxidation and reduction, respectively, due to their lower kinetic stability compared to that of thiophene-fused heptalene derivative **3** reported by Fukazawa et al.

**Table 2:** Redox potentials of heptalenes **6a**, **6b**, and **7**.

Sample	Method	*E*_1_^OX^ [V]	*E*_1_^RED^ [V]	*E*_2_^RED^ [V]
**6a**	CV	+ 0.90 (*E*_pa_)	− 1.84 (*E*_pc_) − 1.23 (*E*_pa_)	–
	(DPV)	(+ 0.76)	(− 1.77)	–
**6b**	CV	+ 0.81 (*E*_pa_)	− 1.87 (*E*_pc_) − 1.25 (*E*_pa_)	–
	(DPV)	(+ 0.72)	(+ 1.80)	–
**7**	CV	+ 0.81 (*E*_pa_)	− 1.85 (*E*_pc_) − 1.31 (*E*_pa_)	− 2.03 (*E*_pc_) − 1.73 (*E*_pa_)
	(DPV)	(+ 0.74)	(− 1.76)	(− 1.94)

V versus Ag/AgNO_3_, 1 mM in benzonitrile containing Et_4_NClO_4_ (0.1 M), Pt electrode (internal diameter: 1.6 mm), scan rate = 100 mVs^−1^ (CV) and 20 mVs^−1^ (DPV), and external standard Fc/Fc^+^ =  + 0.15 V. *E*_pc_ and *E*_pa_ correspond to the cathodic and anodic peak potentials, respectively.

**Figure 7:**
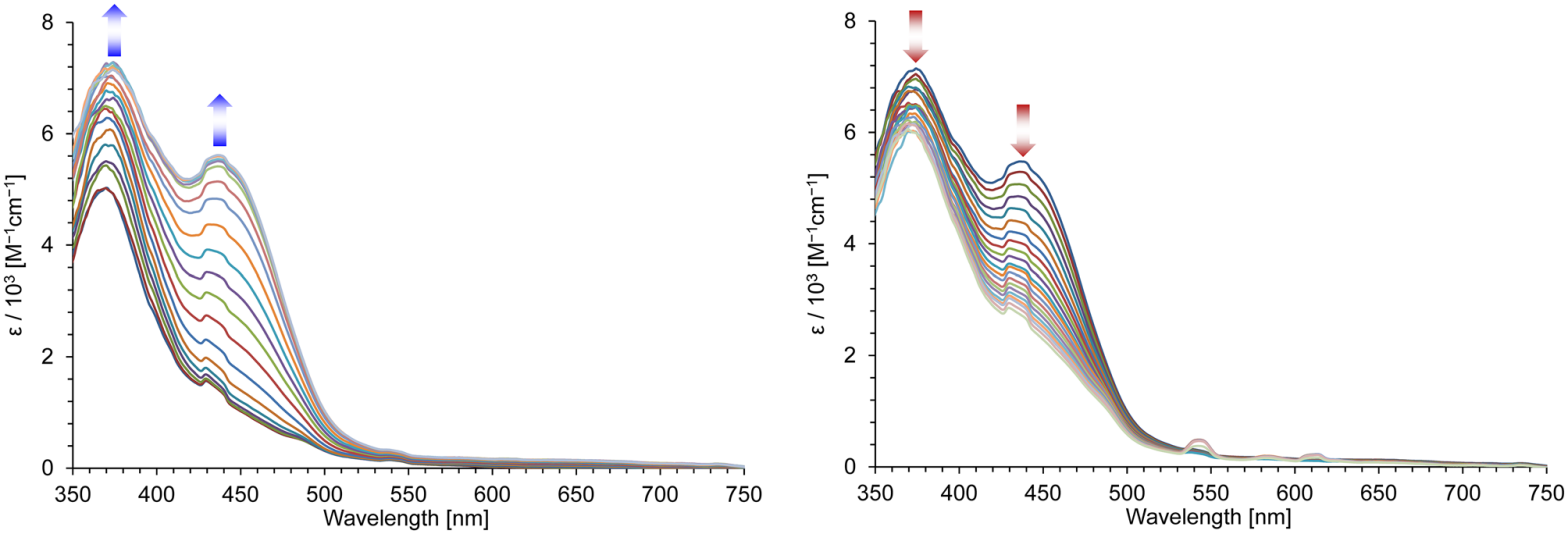
Continuous change in the visible spectrum of **6b**: constant-voltage electrochemical oxidation at + 1.00 V (left) and electrochemical reduction of the oxidized species at ± 0 V (right) in benzonitrile containing Et_4_NClO_4_ (0.1 M) at 30 s intervals.

To observe these unstable cationic and anionic species spectroscopically, spectroelectrochemistry of **6a**, **6b**, and **7** was examined in benzonitrile solution containing Et_4_NClO_4_ (0.1 M) as a supporting electrolyte. We found that the thiophene-fused heptalenes **6a**, **6b**, and **7** exhibited spectral changes under both electrochemical oxidation and reduction conditions. These results suggest the formation of cationic and anionic species by the electrochemical redox reactions. However, the reverse redox reaction of the generated species did not recover the original absorption spectra of **6a**, **6b**, and **7**, completely. This phenomenon indicates that the charged species generated electrochemically are quite unstable as suggested by the irreversibility on the redox waves as observed by the CV measurements. For instance, when the spectral changes of **6b** were measured under the electrochemical oxidation conditions, the development of the peak at λ_max_ = 354 nm was observed, along with the generation of the new absorption band at around 440 nm (Fig. 7, left). However, the reverse reduction of the oxidized species of **6b** did not recover the original spectrum of neutral **6b**, completely (Fig. 7, right).

## Conclusion

In this paper, we described the preparation of thiophene-fused heptalene derivatives **6a**, **6b**, and **7** by the cycloaddition reaction of the corresponding azulenothiophenes with DMAD. The single-crystal X-ray structural analysis of **6a**, **6b**, and **7** revealed their twisted structure, as well as their non-aromatic character denoted by their bond alternation. The measurements of UV/Vis spectra of **6a**, **6b**, and **7** revealed that the fused-thiophene ring directly contributed to the expansion of the conjugated system. In the spectroelectrochemistry measurements, **6a**, **6b**, and **7** exhibited the spectral changes under both electrochemical oxidation and reduction conditions, although the complete recovery of the original absorption spectra was not observed by the reverse redox reactions. These results indicate the generation of labile cationic and anionic species electrochemically from the thiophene-fused heptalene derivatives **6a**, **6b**, and **7**.

## Methods^19,30^

Melting points were determined with a Yanagimoto MPS3 micro melting apparatus and are uncorrected. Voltammetry measurements were carried out with a BAS 100B/W electrochemical workstation equipped with Pt working and auxiliary electrodes and a reference electrode formed from Ag/AgNO_3_ (0.01 M) in acetonitrile containing tetrabutylammonium perchlorate (0.1 M). High-resolution mass spectra were obtained with a Bruker Daltonics APEX III instrument (dithranol as a matrix substance and/or CF_3_CO_2_Ag as an auxiliary agent). IR and UV/Vis spectra were measured with JASCO FT/IR-4100 and Shimadzu UV-2550 spectrophotometers. ^1^H and ^13^C NMR spectra were recorded in CDCl_3_ with a JEOL ECA500 at 500 MHz and 125 MHz, respectively.

### Synthesis of 6a

A solution of **4a** (800 mg, 4.34 mmol) and DMAD (925 mg, 6.51 mmol) in tetralin (20 mL) was stirred at 200 °C for 45 min under an Ar atmosphere. After the reaction, the crude product was purified by alumina column chromatography with AcOEt as an eluent and reversed-phase chromatography with 80% MeOH to afford **6b** (843 mg, 2.58 mmol, 60%) as reddish-orange crystals.

IR (AT-IR): ν_max_ = 3,094 (w), 2,954 (w), 1717 (m), 1701 (s), 1,630 (w), 1603 (w), 1572 (w), 1,540 (w), 1507 (w), 1,437 (m), 1,405 (w), 1,387 (w), 1,349 (w), 1,290 (m), 1,262 (s), 1,246 (m), 1,206 (m), 1,157 (w), 1,120 (m), 1,103 (w), 1,060 (w), 1,034 (w), 1,007 (w), 984 (w), 948 (w), 933 (w), 903 (w), 889 (w), 865 (w), 840 (w), 805 (w), 792 (w), 775 (m), 752 (w), 731 (w), 721 (m), 693 (w), 666 (w), 653 (m) cm^−1^; UV/Vis (CH_2_Cl_2_): λ_max_ = 289 (4.39), 351 (3.61) nm; ^1^H NMR (500 MHz, CDCl_3_): δ_H_ = 7.83 (s, 1H), 7.22 (d, *J* = 5.2 Hz, 1H), 6.94 (d, *J* = 5.2 Hz, 1H), 6.52 − 6.51 (m, 2H), 6.31 − 6.28 (m, 1H), 6.17 (t, *J* = 3.6 Hz, 1H), 5.92 (d, *J* = 10.9 Hz, 1H), 3.75 (s, 3H, CO_2_Me), 3.67 (s, 3H, CO_2_Me) ppm; ^13^C NMR (125 MHz, CDCl_3_): δ_C_ = 167.70, 167.44, 143.84, 142.49, 137.67, 137.15, 133.16, 132.20, 131.15, 130.71, 130.00, 129.62, 126.68, 126.47, 125.21, 124.65, 52.18, 51.68 ppm; HRMS (MALDI-TOF, positive): calcd for C_18_H_14_O_4_S^+^ [M]^+^ 326.0607, found: 326.0595, C_18_H_14_O_4_S + Ag^+^ [M + Ag]^+^ 432.9658, found: 432.9686.

### Synthesis of 6b

A solution of **4b** (450 mg, 1.99 mmol) and DMAD (424 mg, 2.99 mmol) in tetralin (20 mL) was stirred at 200 °C for 20 min under an Ar atmosphere. After the reaction, the crude product was purified by alumina column chromatography with AcOEt as an eluent and reversed-phase chromatography with 80% MeOH to afford **6b** (398 mg, 1.08 mmol, 54%) as reddish-orange crystals. M.p. 148 − 149 °C; IR (AT-IR): ν_max_ = 3,108 (w), 2,956 (w), 1699 (s), 1578 (w), 1,435 (m), 1,365 (w), 1,299 (m), 1,279 (s), 1,260 (s), 1,236 (m), 1,215 (w), 1,191 (m), 1,169 (m), 1,120 (m), 1,092 (w), 1,058 (m), 1,034 (w), 1,003 (w), 964 (w), 950 (w), 902 (w), 891 (w), 873 (w), 854 (w), 840 (w), 802 (m), 787 (s), 769 (m), 752 (m), 739 (s), 730 (s), 678 (m), 661 (w) cm^−1^; UV/Vis (CH_2_Cl_2_): λ_max_ = 289 (4.40), 354 (3.62) nm; ^1^H NMR (500 MHz, CDCl_3_): δ_H_ = 7.81 (s, 1H), 7.20 (d, *J* = 5.4 Hz, 1H), 6.95 (d, *J* = 5.4 Hz, 1H), 6.45 − 6.52 (m, 2H), 6.09 (d, *J* = 5.7 Hz, 1H), 5.72 (s, 1H), 3.75 (s, 3H, CO_2_Me), 3.67 (s, 3H, CO_2_Me), 2.56 (sept, *J* = 6.9 Hz, 1H, *i-*Pr), 1.15 (d, *J* = 6.9 Hz, 6H, *i-*Pr) ppm; ^13^C NMR (125 MHz, CDCl_3_): δ_C_ = 167.96, 167.87, 149.64, 143.44, 142.19, 137.08, 136.89, 134.50, 132.25, 130.41, 129.64, 129.01, 128.06, 126.15, 124.54, 120.99, 52.17, 51.59, 35.74, 22.53 ppm; HRMS (MALDI-TOF, positive): calcd for C_21_H_20_O_4_S^+^ [M]^+^ calcd: 368.1077, found: 368.1098, C_18_H_14_O_4_S + Ag^+^ [M + Ag]^+^ calcd: 475.0128, found: 475.0142.

### Synthesis of 7

A solution of **5** (500 mg, 2.72 mmol) and DMAD (578 mg, 4.07 mmol) in tetralin (20 mL) was stirred at 200 °C for 45 min under an Ar atmosphere. After the reaction, the crude product was purified by alumina column chromatography with AcOEt as an eluent and reversed-phase chromatography with 80% MeOH to afford **6b** (208 mg, 0.638 mmol, 23%) as reddish-orange crystals. M.p. 159 − 161 °C; IR (AT-IR): ν_max_ = 3,108 (w), 2,956 (w), 1699 (s), 1578 (w), 1,435 (m), 1,365 (w), 1,299 (m), 1,279 (s), 1,260 (s), 1,236 (m), 1,215 (w), 1,191 (m), 1,169 (m), 1,120 (m), 1,092 (w), 1,058 (m), 1,034 (w), 1,003 (w), 964 (w), 950 (w), 902 (w), 891 (w), 873 (w), 854 (w), 840 (w), 802 (m), 787 (s), 769 (m), 752 (m), 739 (s), 730 (s), 678 (m), 661 (w) cm^−1^; UV/Vis (CH_2_Cl_2_): λ_max_ = 293 (4.48), 354 (3.65) nm; ^1^H NMR (500 MHz, CDCl_3_): δ_H_ = 7.85 (s, 1H), 7.57 (d, *J* = 5.2 Hz, 1H), 6.70 (d, *J* = 5.2 Hz, 1H), 6.56 (dd, *J* = 11.0, 6.3 Hz, 1H), 6.50 (dd, *J* = 11.0, 6.3 Hz, 1H), 6.30 (dd, *J* = 11.0, 6.3 Hz, 1H), 5.99 (d, *J* = 6.6 Hz, 1H), 5.93 (d, *J* = 11.2 Hz, 1H), 3.75 (s, 3H, CO_2_Me), 3.67 (s, 3H, CO_2_Me) ppm; ^13^C NMR (125 MHz, CDCl_3_): δ_C_ = 167.45, 167.41, 144.07, 143.91, 136.92, 134.99, 132.75, 132.42, 131.91, 131.61, 130.40, 129.58, 128.95, 128.82, 125.24, 124.38, 52.23, 51.66 ppm; HRMS (MALDI-TOF, positive): calcd for C_18_H_14_O_4_S^+^ [M]^+^ calcd: 326.0607, found: 326.0580, C_18_H_14_O_4_S + Ag^+^ [M + Ag]^+^ calcd: 432.9658, found: 432.9679.

## Supplementary information

Supplementary Information.
