# Supplementary material for: Synthesis of thiophene-fused heptalenes by cycloaddition of azulenothiophenes with dimethyl acetylenedicarboxylate
Source: Sci Rep. 2020 Jul 27;10:12477. doi: 10.1038/s41598-020-69425-w (PMC7385116; doi:10.1038/s41598-020-69425-w)
Supplement: Supplementary file 1 — Supplementary Information. [file 41598_2020_69425_MOESM1_ESM.pdf]

# *Supporting Information*

## Synthesis of Thiophene-fused Heptalenes by Cycloaddition of Azulenothiophenes with Dimethyl Acetylenedicarboxylate

Taku Shoji,*[1] Kota Miura,[1] Yukino Ariga,[1] Akari Yamazaki,[1] Shunji Ito,[2] and Masafumi Yasunami[3]

[1] Department of Material Science, Graduate School of Science and Technology, Shinshu University, Matsumoto 390-8621, Nagano, Japan.

[2] Graduate School of Science and Technology, Hirosaki University, Hirosaki 036-8561, Aomori, Japan.

[3] Department of Chemical Biology and Applied Chemistry, College of Engineering, Nihon University, Koriyama 963-8642, Fukushima, Japan.

### ➢ *Contents*

1. Copies of $^1$H NMR, $^{13}$C NMR, COSY and HRMS of reported compounds. S1–S6
2. UV/Vis and fluorescent spectra of **6a,b** and **7**. S7–S9
3. Cyclic voltammograms and spectroelectrochemistry of **6a,b** and **7**. S10–S12
4. ORTEP Drawing of **6a,b** and **7**. S13–S14

1. **Copies of $^{1}$H NMR, $^{13}$C NMR, COSY and HRMS of reported compounds (Figures S1–S12).**

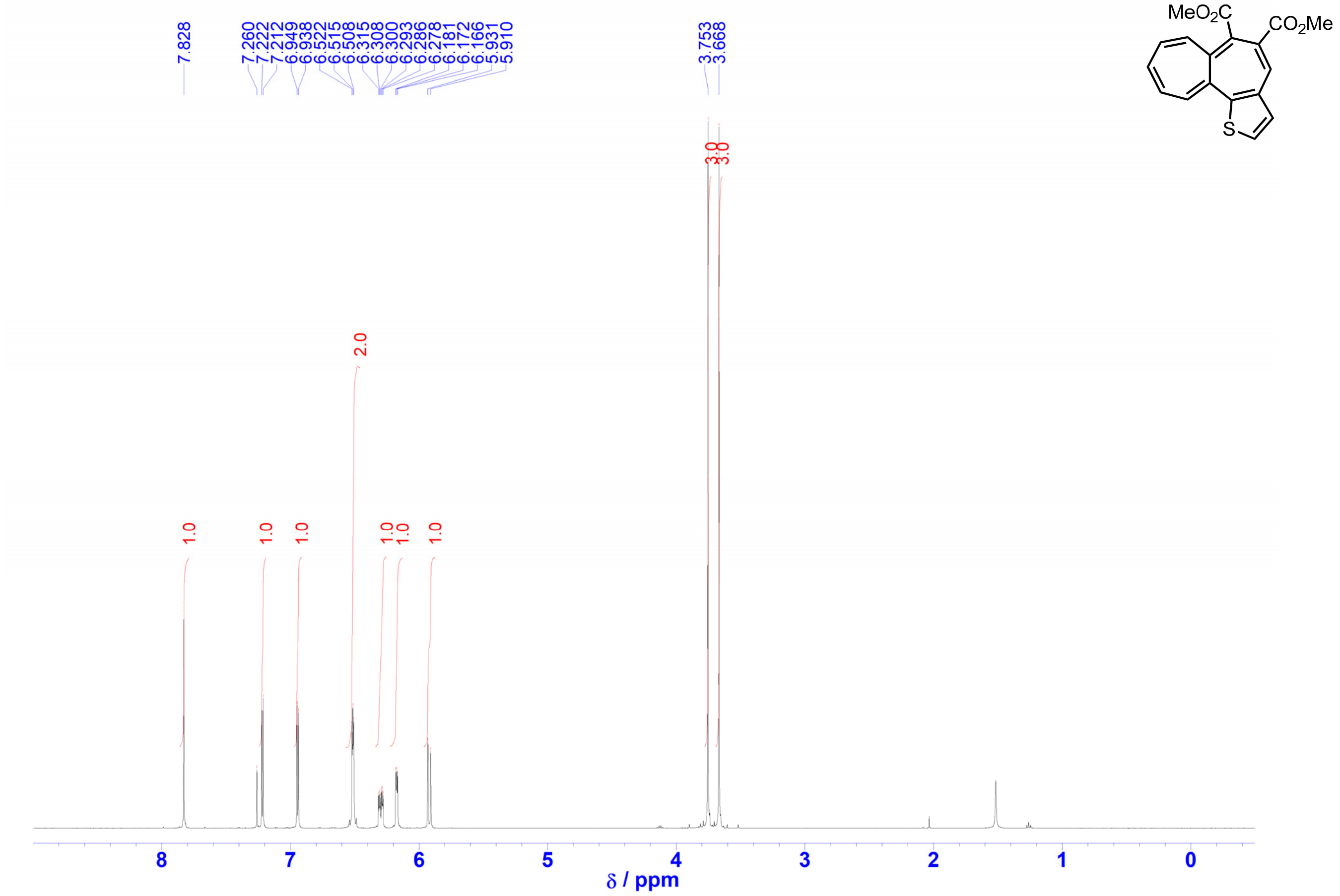


**Figure S1.** $^{1}$H NMR spectrum of **6a** in $CDCl_3$ (500 MHz).

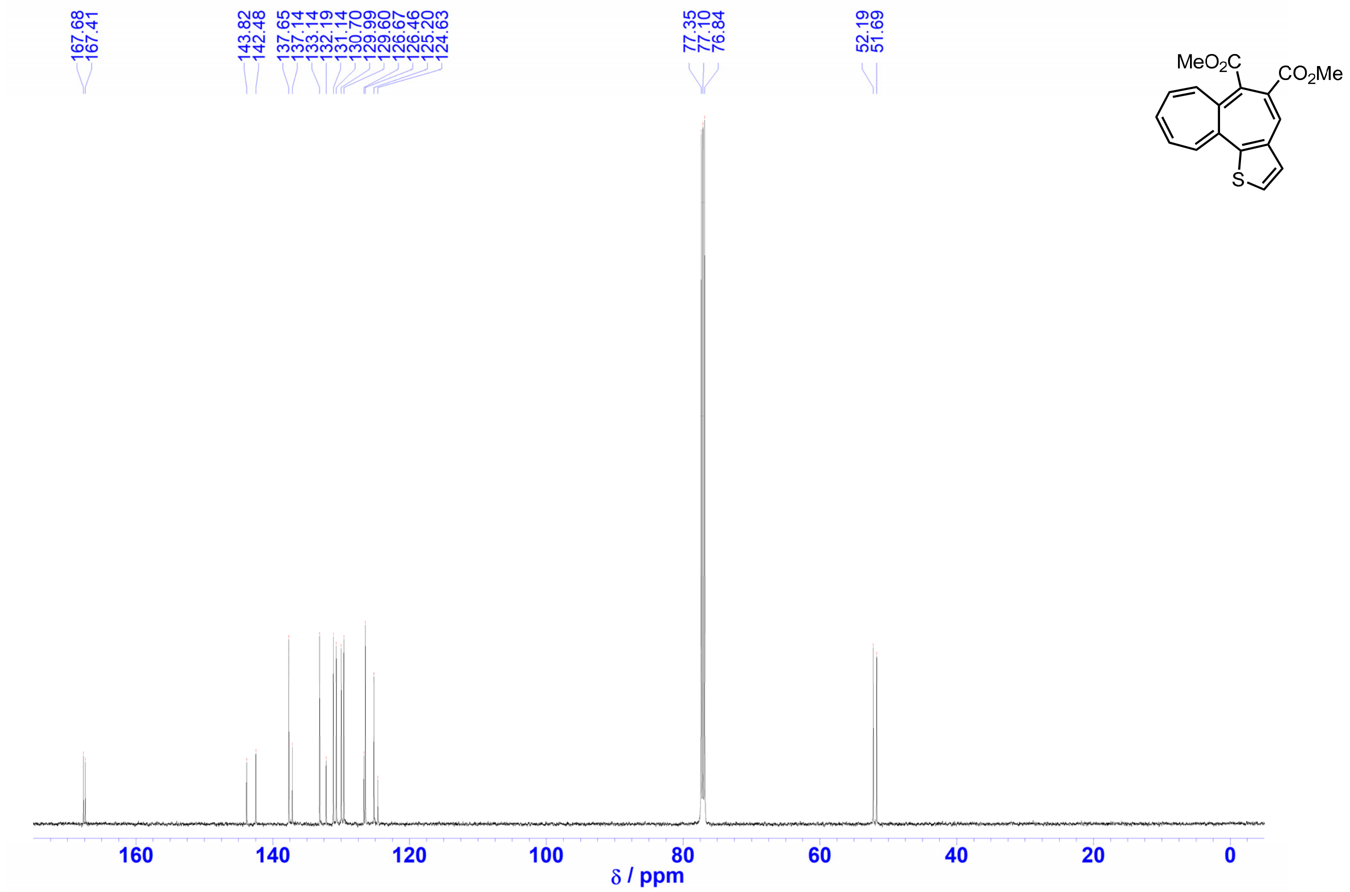


**Figure S2.** $^{13}$C NMR spectrum of **2a** in $CDCl_3$ (125 MHz).

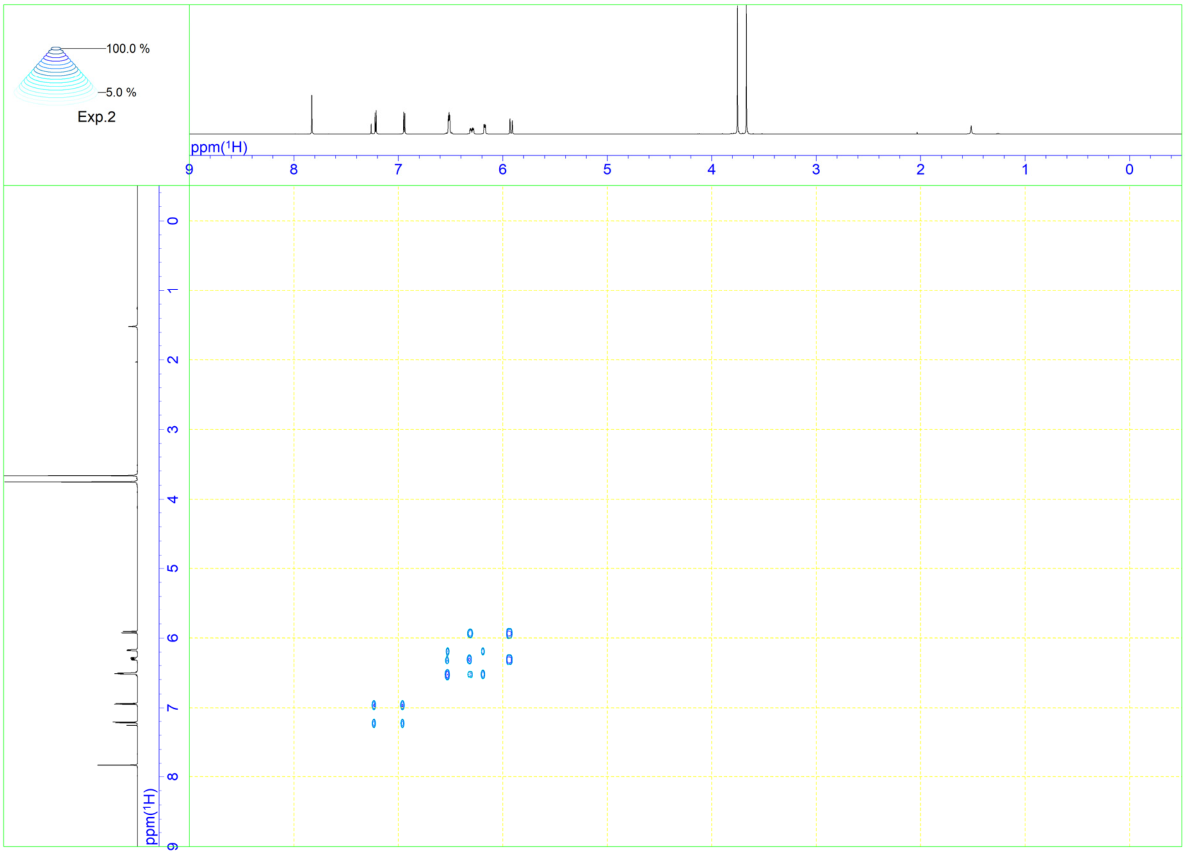


**Figure S3.** COSY spectrum of **6a** in $CDCl_3$ (500 MHz).

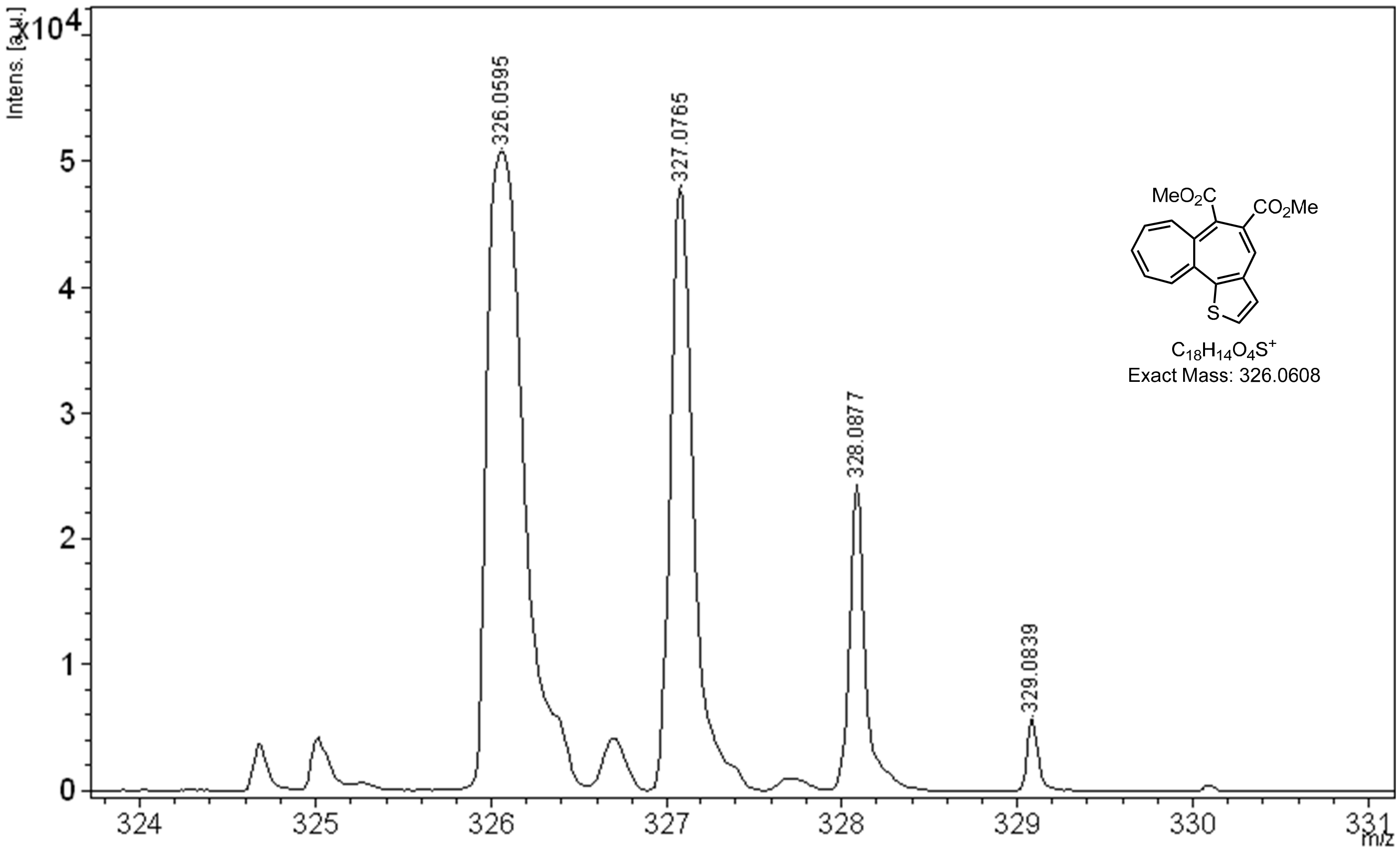


**Figure S4.** HRMS (MALDI-TOF, positive) of **6a.**

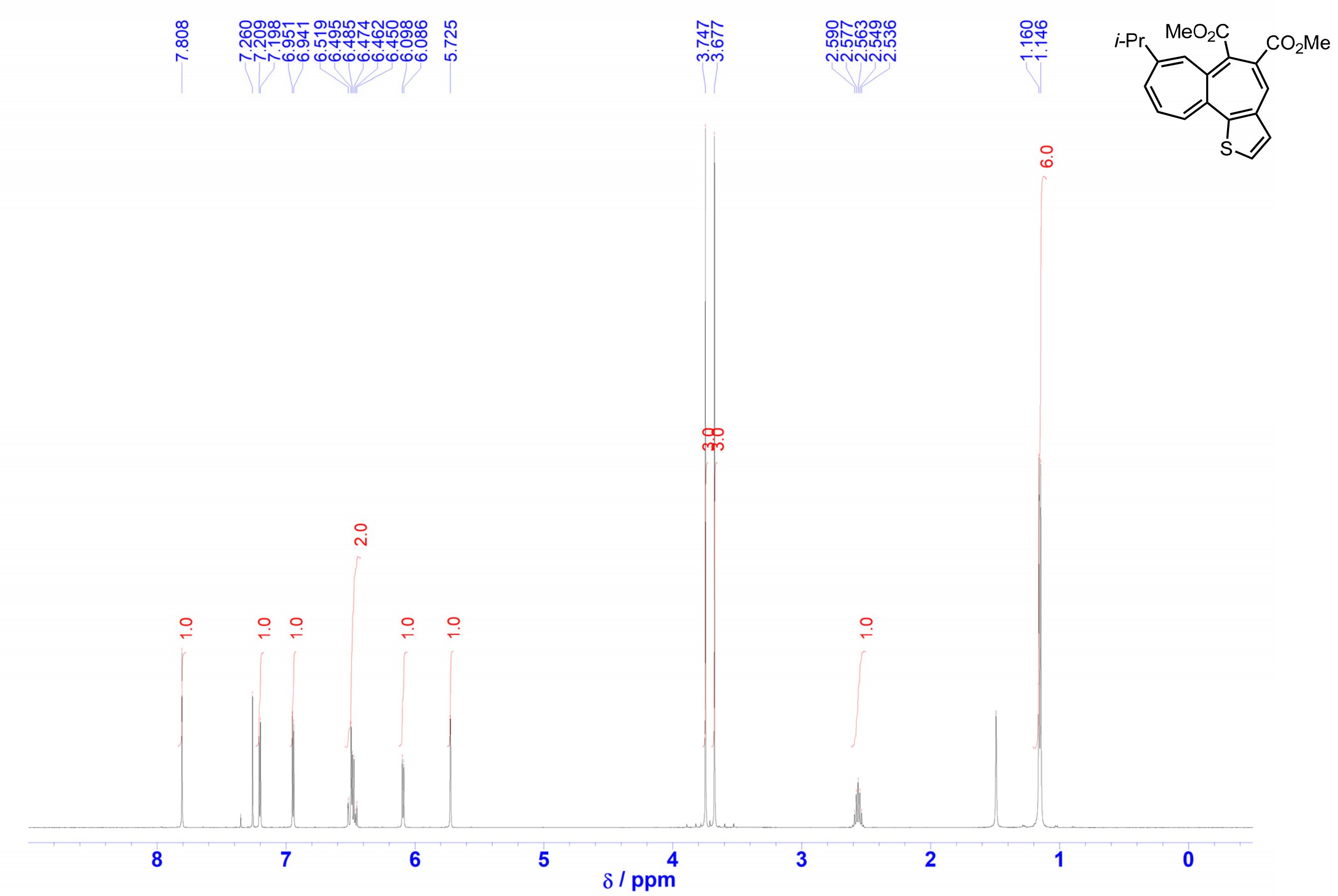


**Figure S5.** $^1$H NMR spectrum of **6b** in $CDCl_3$ (500 MHz).

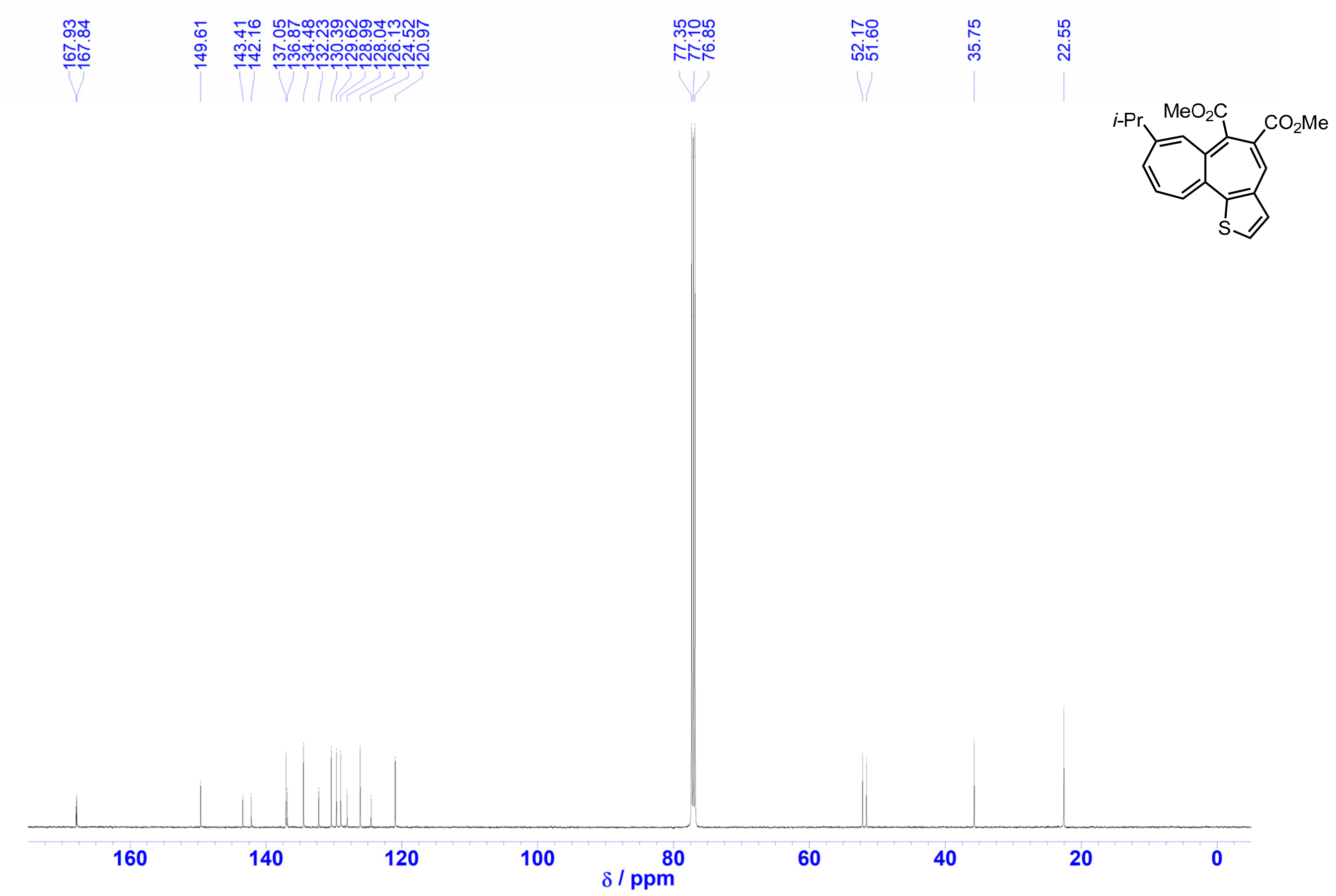


**Figure S6.** $^{13}$C NMR spectrum of **6b** in $CDCl_3$ (125 MHz).

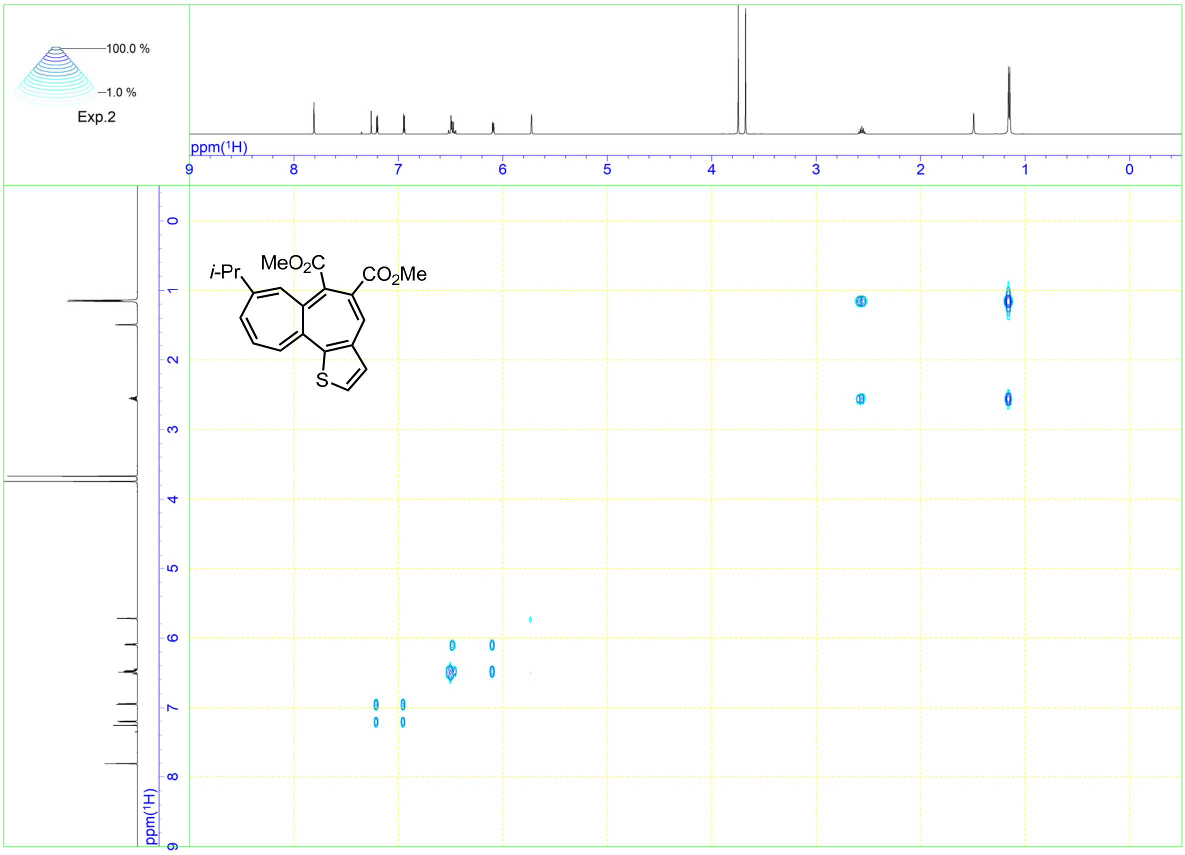


**Figure S7.** COSY spectrum of **6b** in $CDCl_3$ (500 MHz).

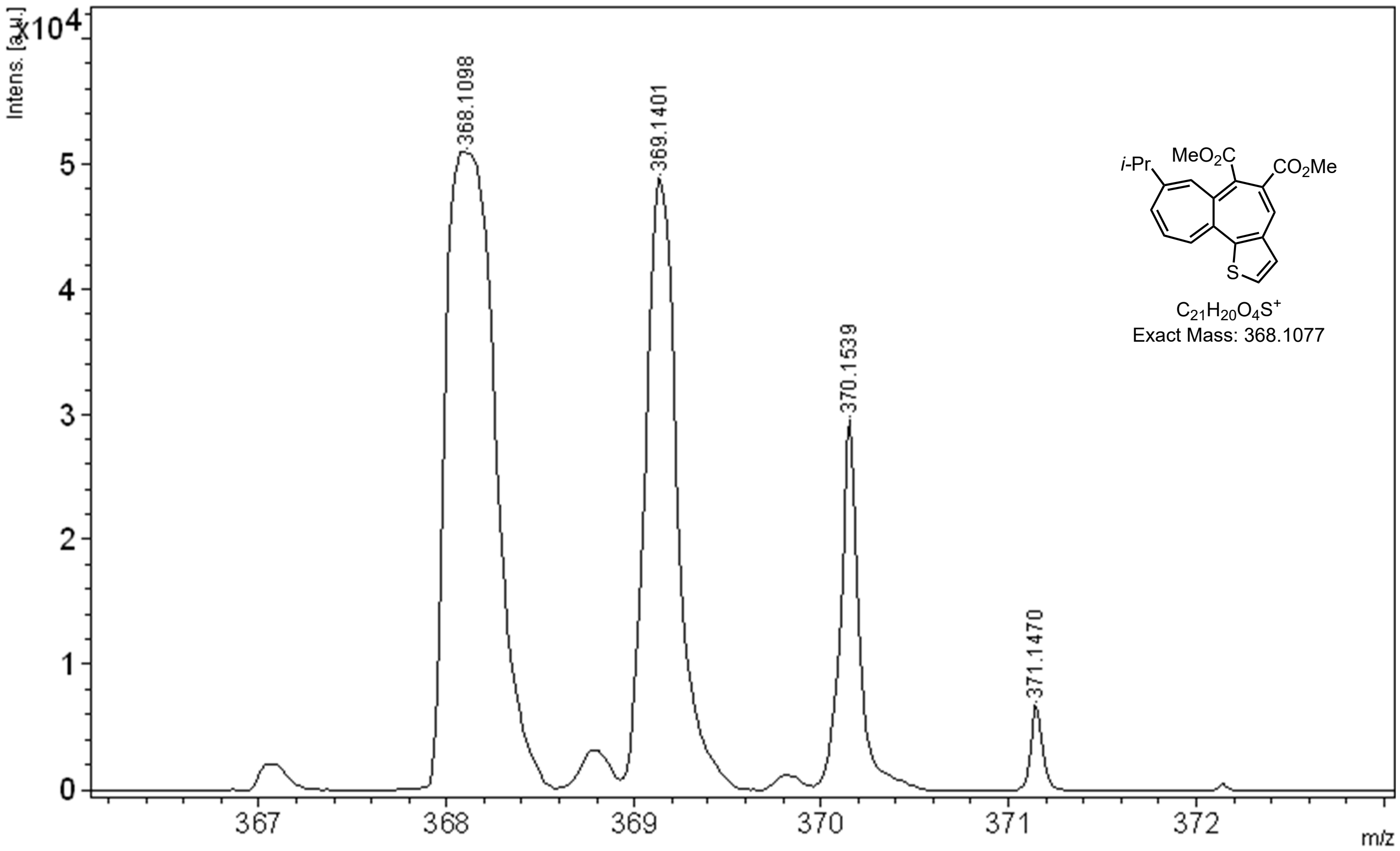


**Figure S8.** HRMS (MALDI-TOF, positive) of **6b**.

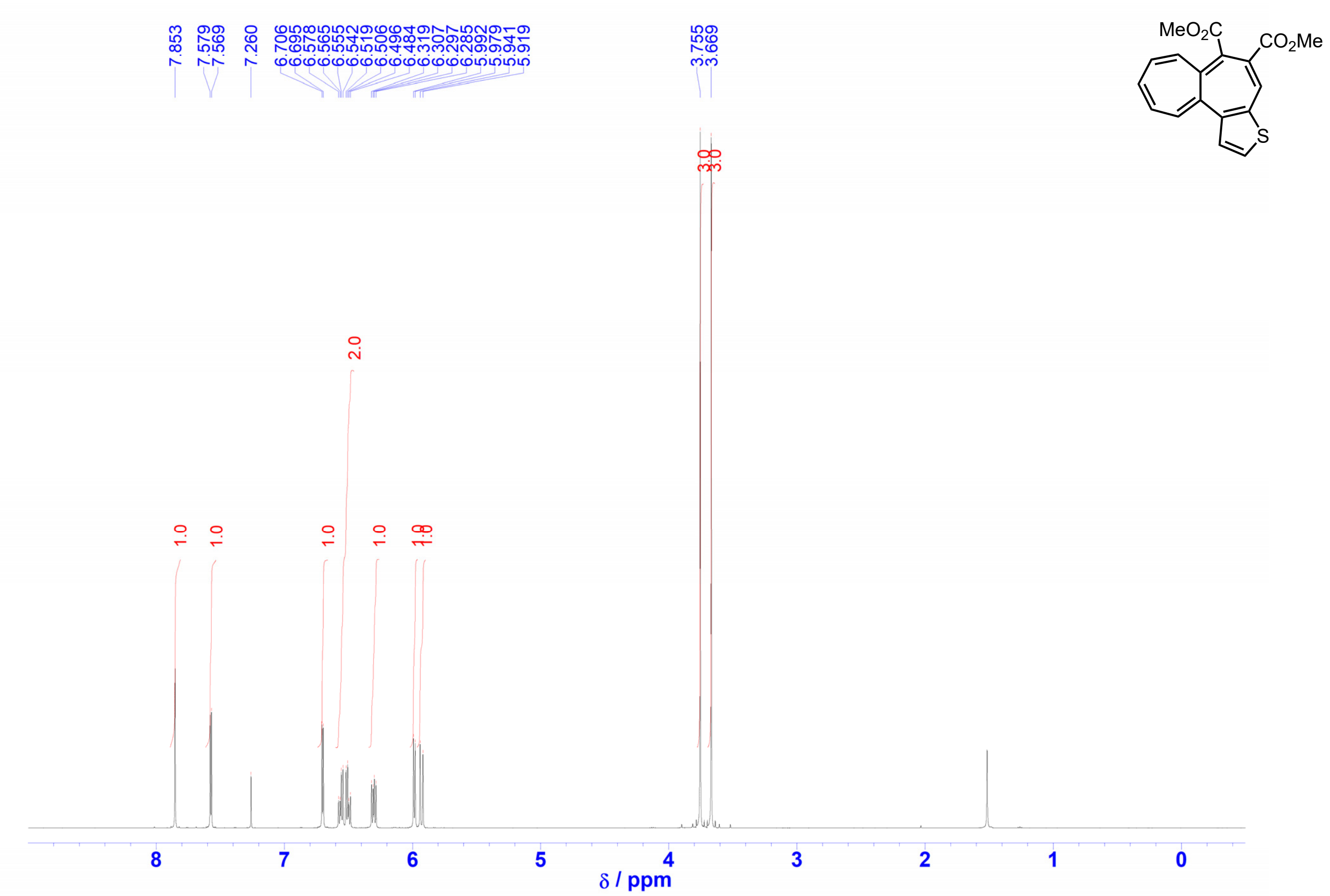


**Figure S9.** $^{1}H$ NMR spectrum of **7** in $CDCl_3$ (500 MHz).

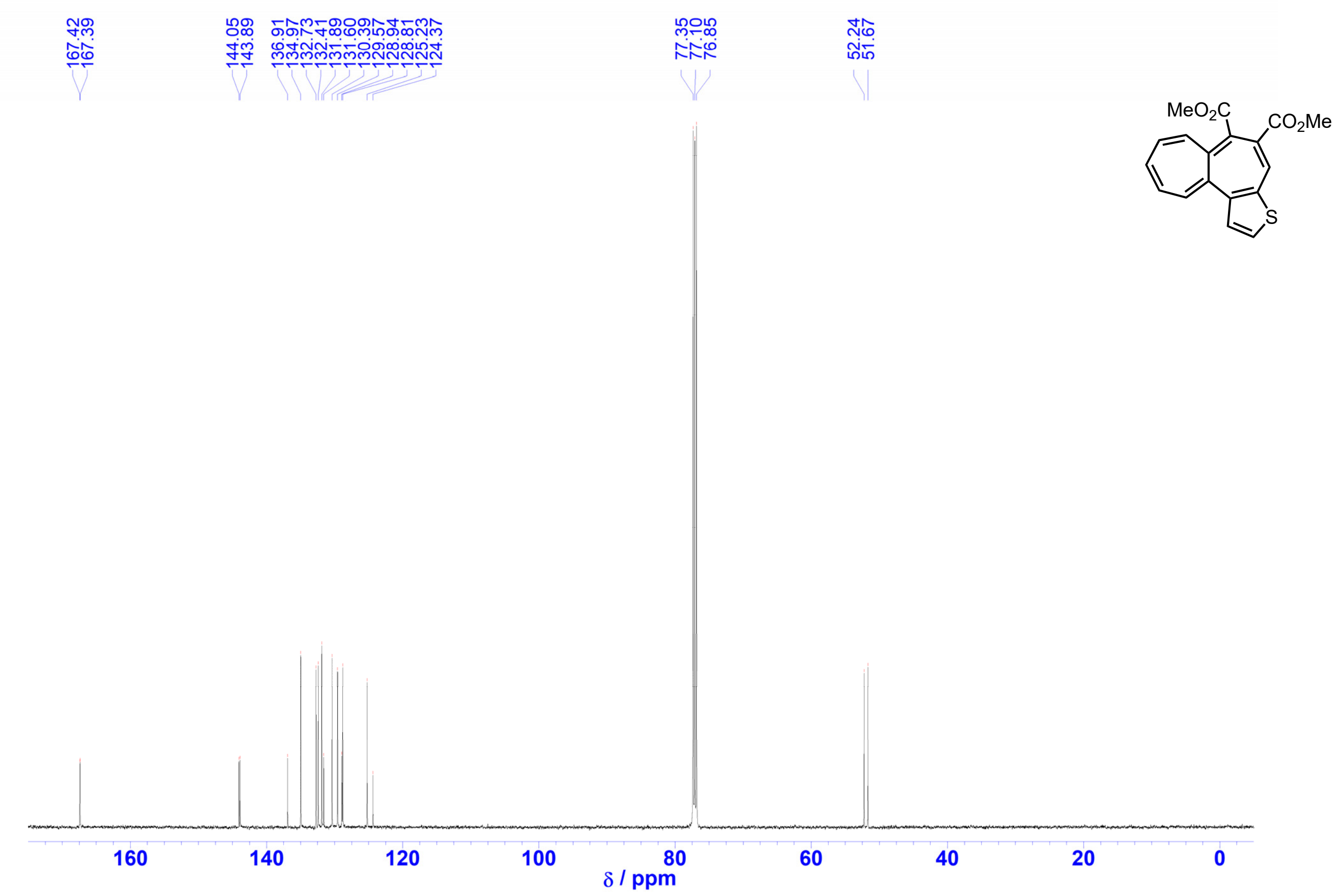


**Figure S10.** $^{13}C$ NMR spectrum of **7** in $CDCl_3$ (125 MHz).

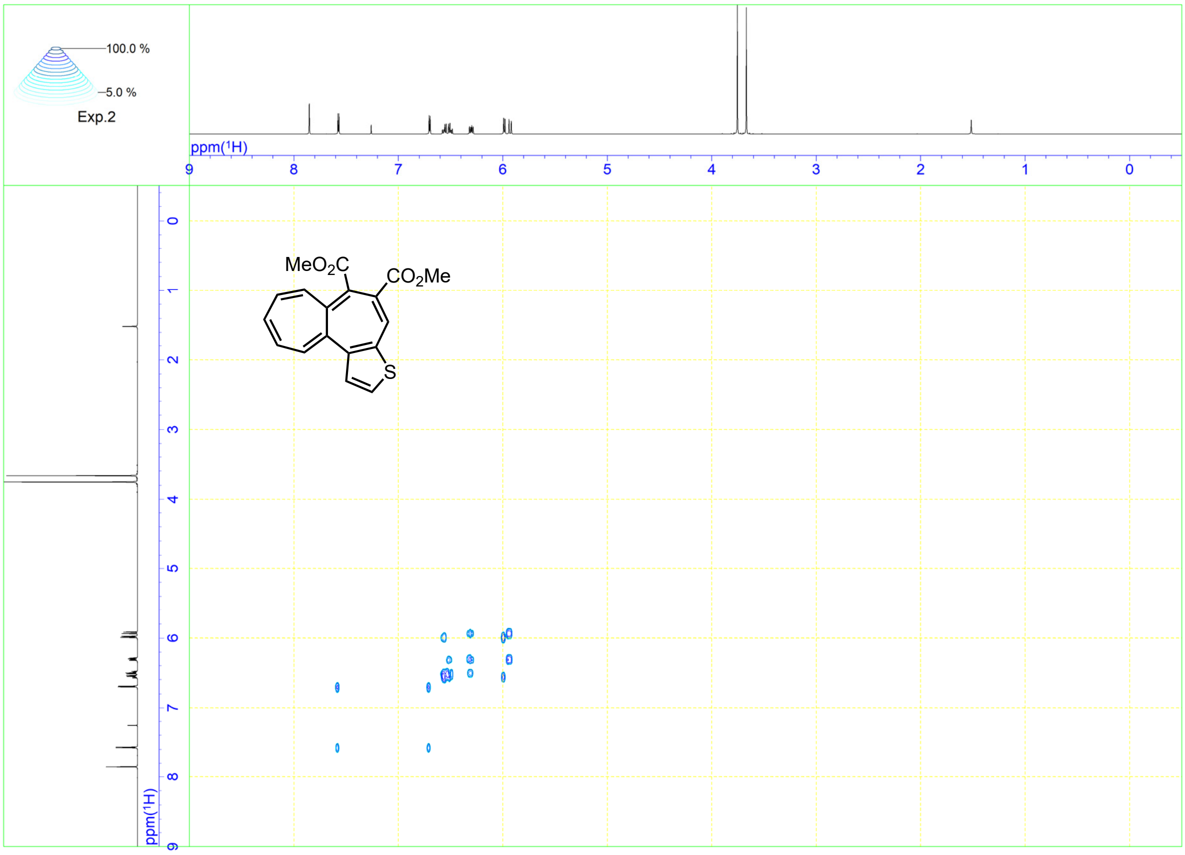


**Figure S11.** COSY spectrum of **7** in $CDCl_3$ (500 MHz).

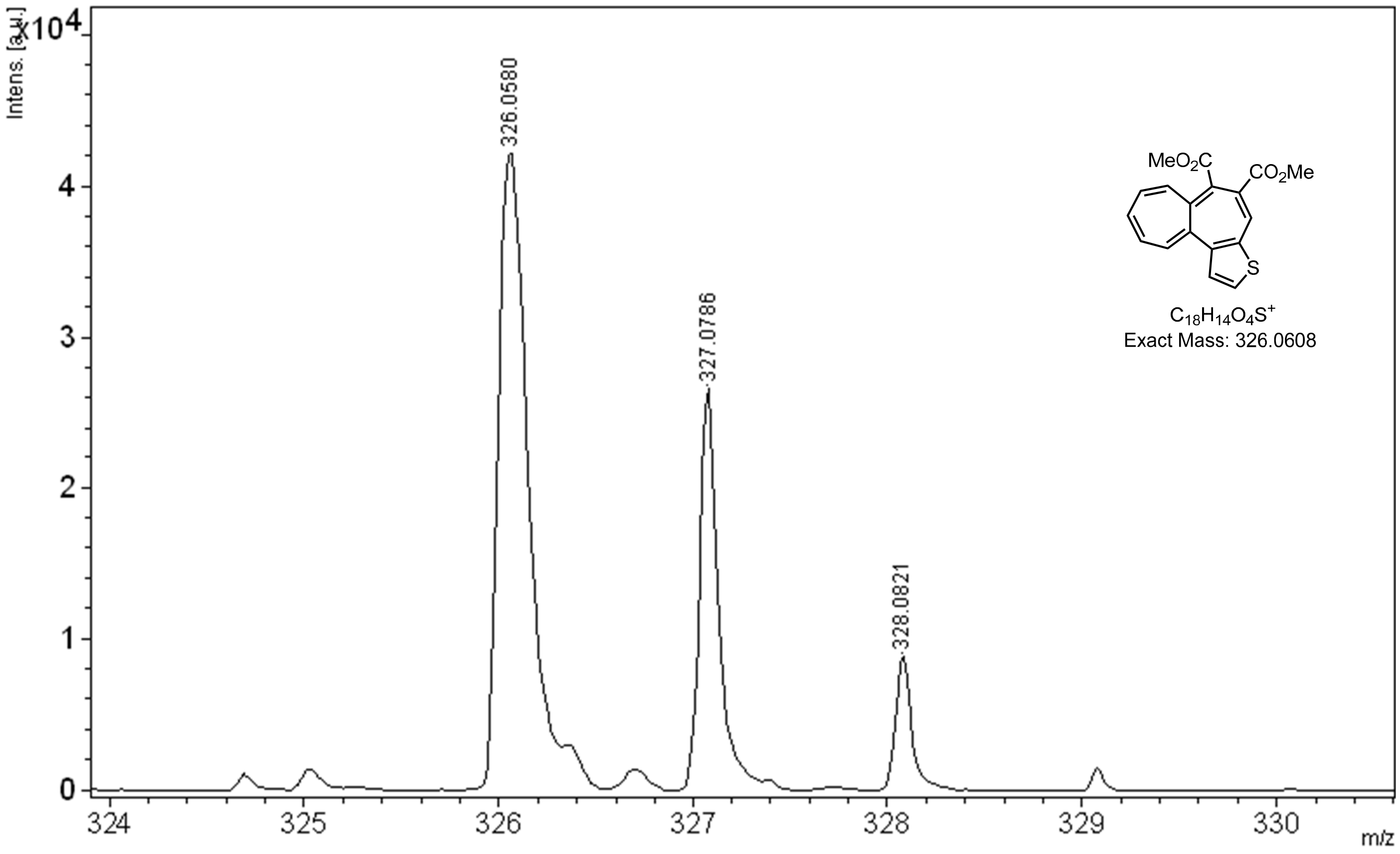


**Figure S12.** HRMS (MALDI-TOF, positive) of **7**.

## 2. UV/Vis and fluorescent spectra of 6a,b and 7 (Figures S13–S18).

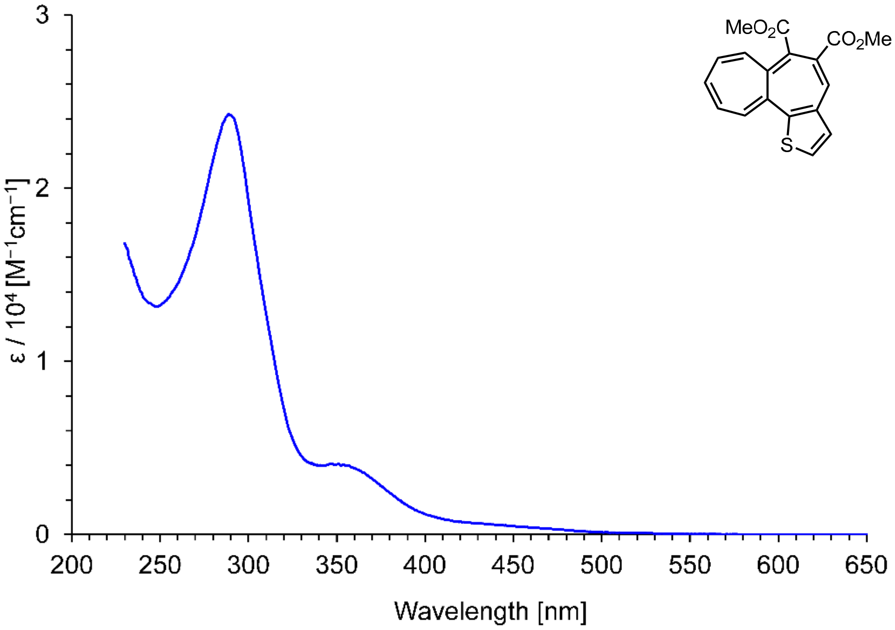


**Figure S13.** UV/Vis spectra of **6a** in $CH_2Cl_2$.

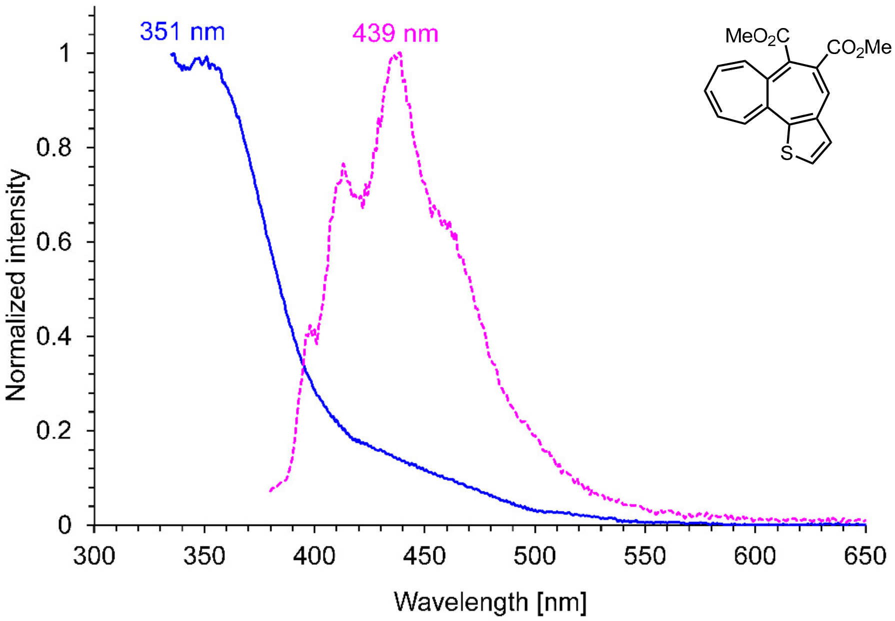


**Figure S14.** UV/Vis spectrum (blue line) and fluorescent spectrum (pink dotted-line) of **6a** in $CH_2Cl_2$.

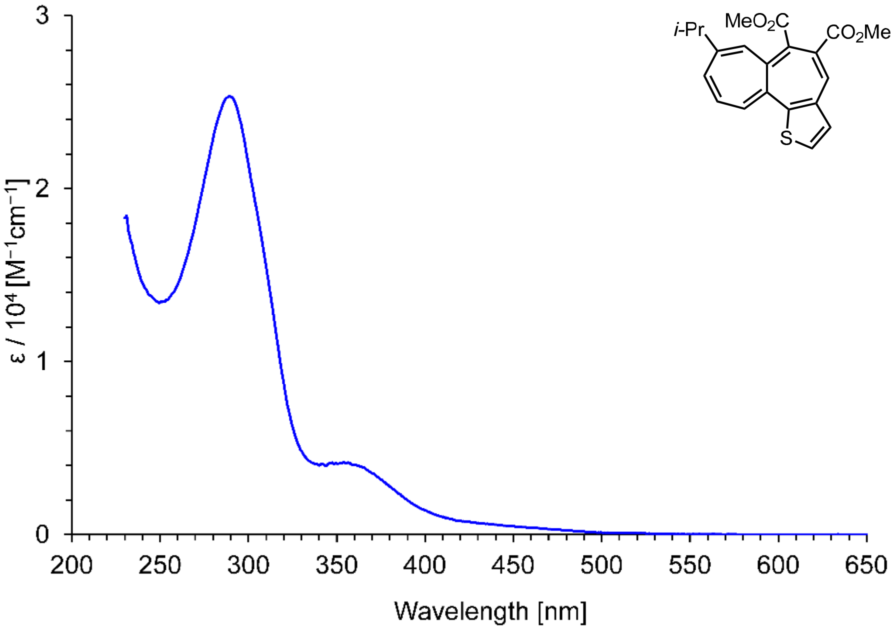


**Figure S15.** UV/Vis spectra of **6b** in $CH_2Cl_2$.

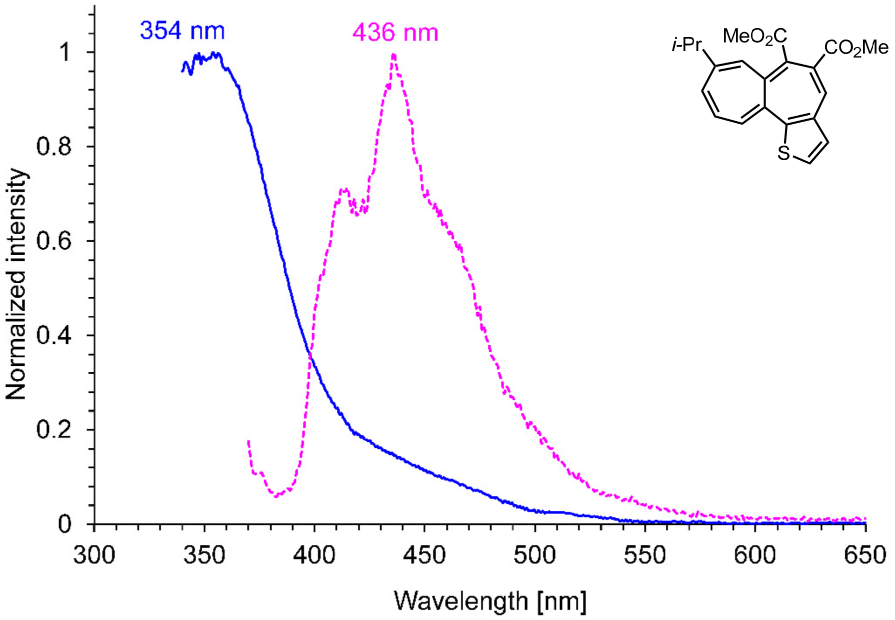


**Figure S16.** UV/Vis spectrum (blue line) and fluorescent spectrum (pink dotted-line) of **6b** in $CH_2Cl_2$.

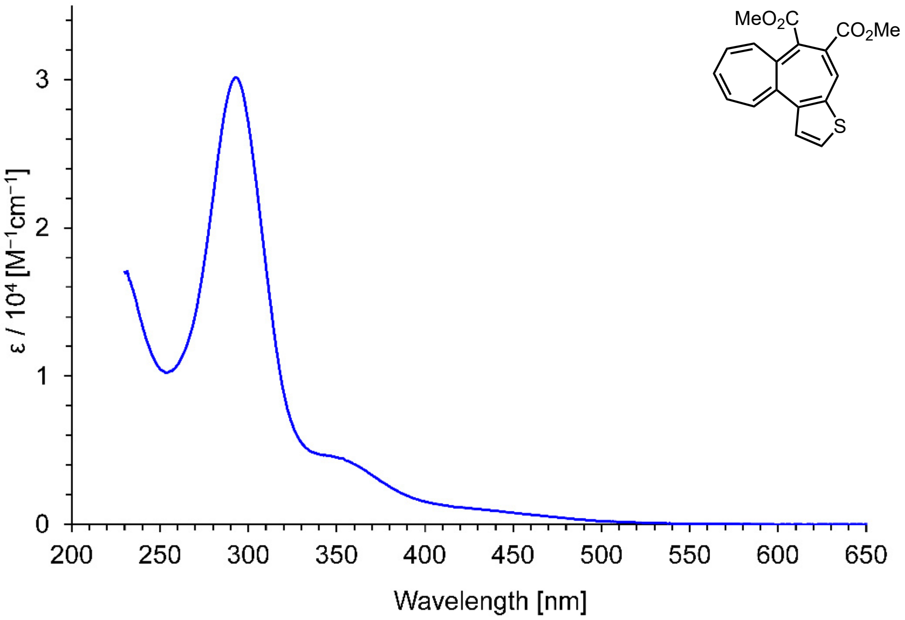


**Figure S17.** UV/Vis spectra of **7** in $CH_2Cl_2$.

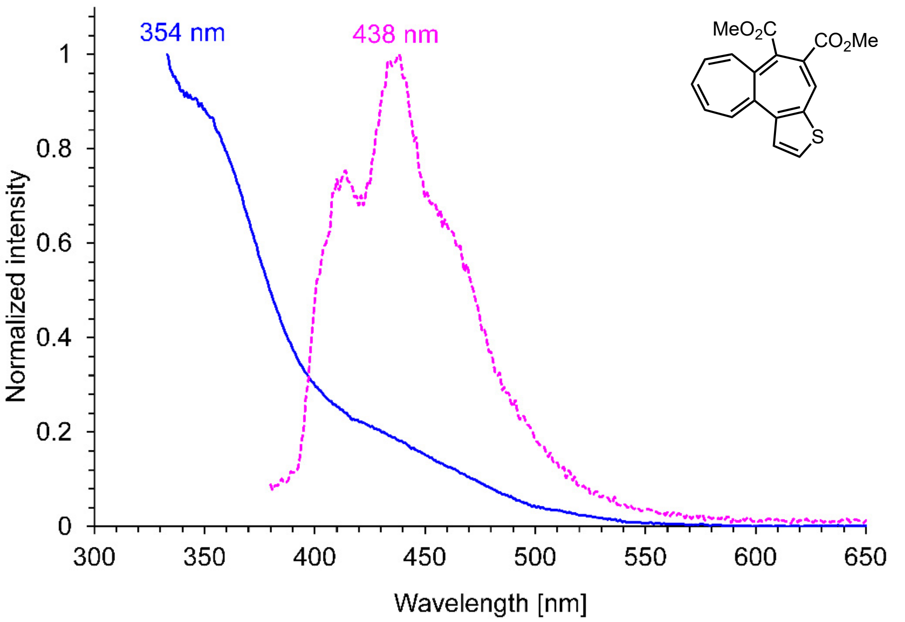


**Figure S18.** UV/Vis spectrum (blue line) and fluorescent spectrum (pink dotted-line) of **7** in $CH_2Cl_2$.

## 3. UV/Vis and fluorescent spectra of 6a,b and 7 (Figures S19−S26).

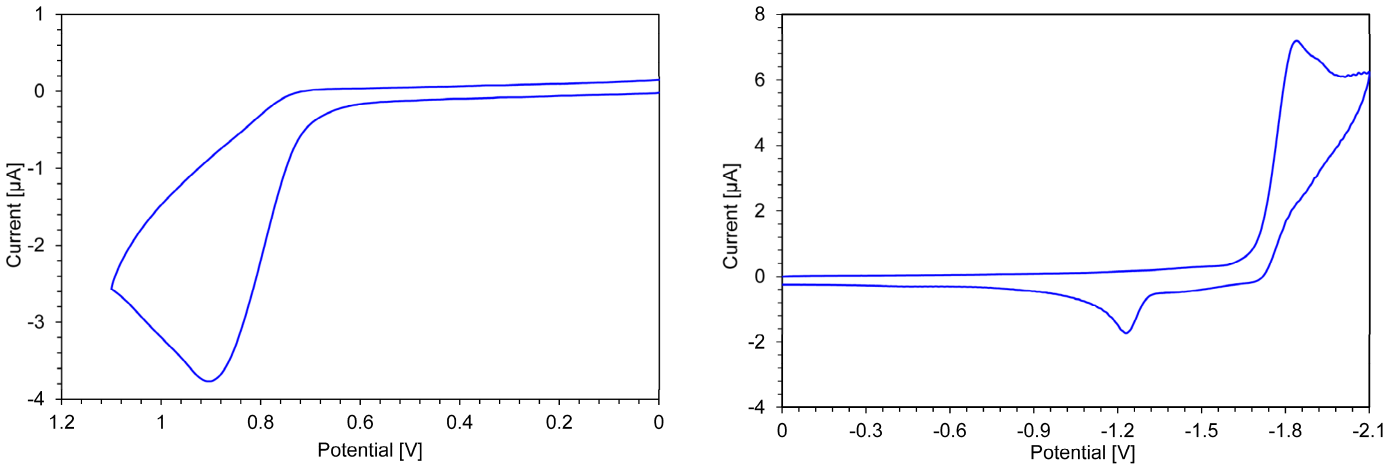


**Figure S19.** Cyclic voltammogram for oxidation (left) and reduction (right) of **6a** (1 mM) in benzonitrile containing $Et_4NClO_4$ (0.1 M) as the supporting electrolyte; scan rate = 100 $mVs^{-1}$.

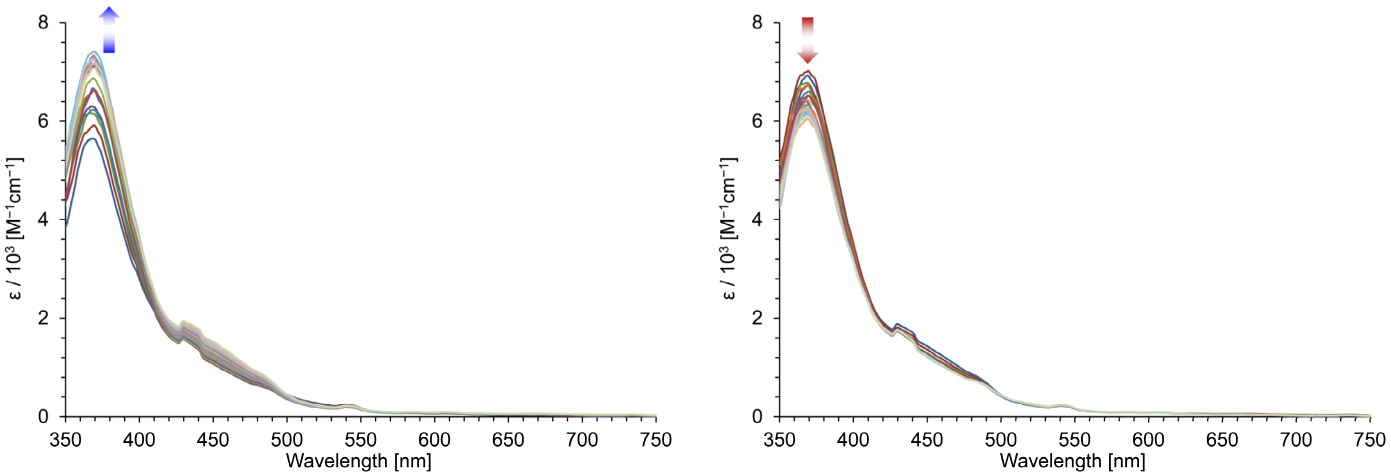


**Figure S20.** Continuous change in the visible spectrum of **6a**: constant-voltage electrochemical oxidation at +1.10 V (left) and electrochemical reduction of the oxidized species at ±0 V (right) in benzonitrile containing $Et_4NClO_4$ (0.1 M) at 30 sec intervals.

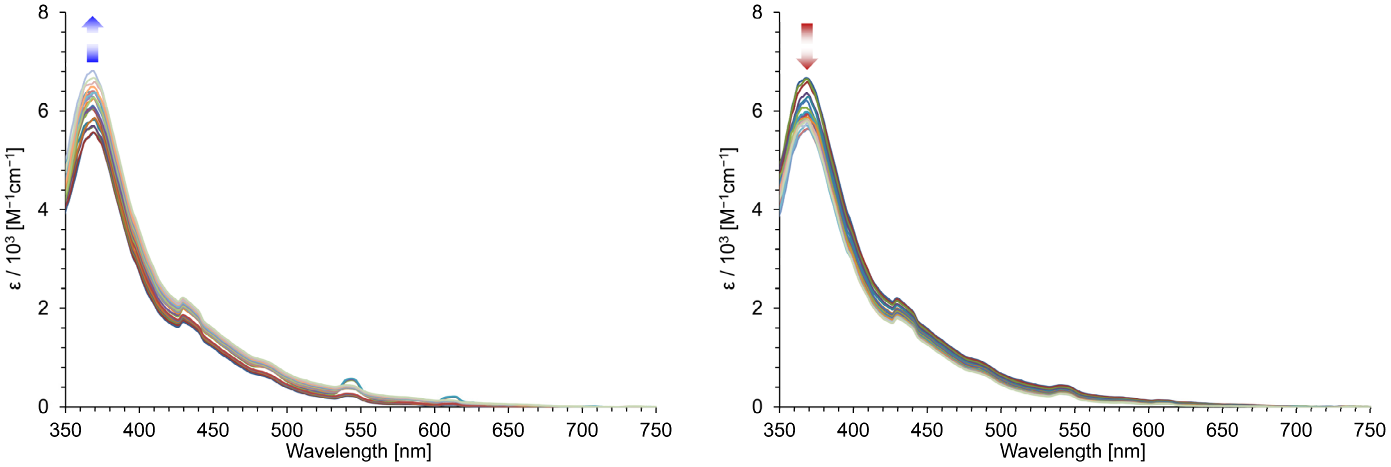


**Figure S21.** Continuous change in the visible spectrum of **6a**: constant-voltage electrochemical reduction at −2.10 V (left) and electrochemical oxidation of the reduced species at ±0 V (right) in benzonitrile containing $Et_4NClO_4$ (0.1 M) at 30 sec intervals.

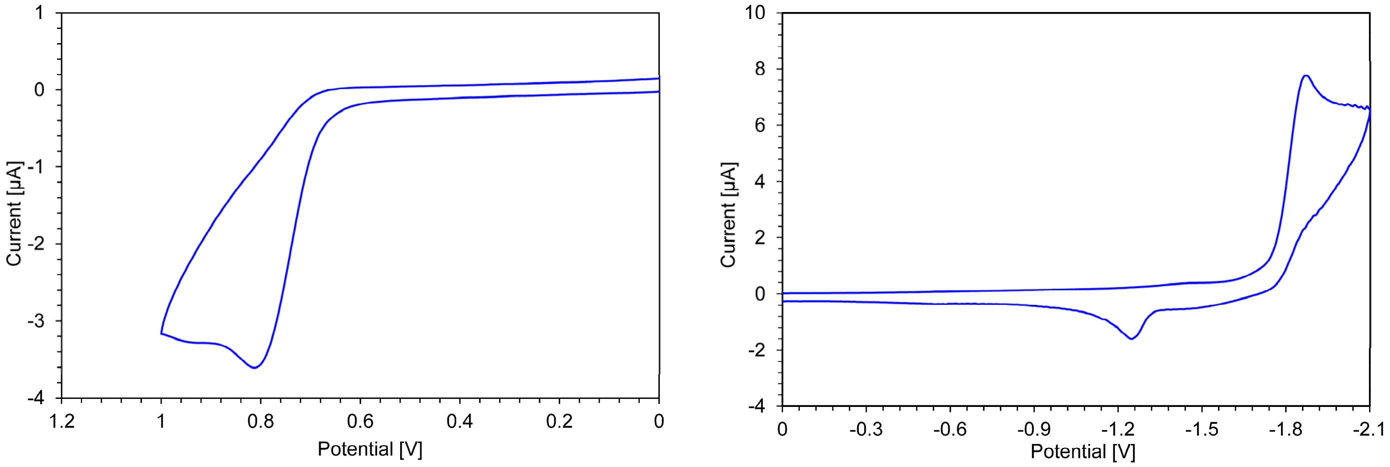


**Figure S22.** Cyclic voltammogram for oxidation (left) and reduction (right) of **6b** (1 mM) in benzonitrile containing $Et_4NClO_4$ (0.1 M) as the supporting electrolyte; scan rate = 100 $mVs^{-1}$.

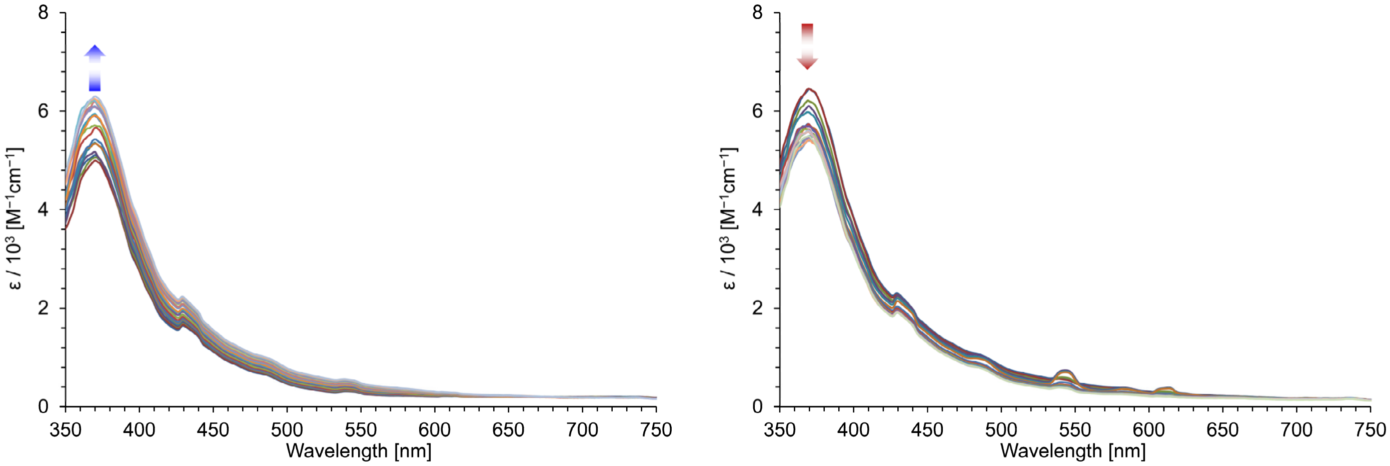


**Figure S23.** Continuous change in the visible spectrum of **6b**: constant-voltage electrochemical reduction at −2.10 V (left) and electrochemical oxidation of the reduced species at ±0 V (right) in benzonitrile containing $Et_4NClO_4$ (0.1 M) at 30 sec intervals.

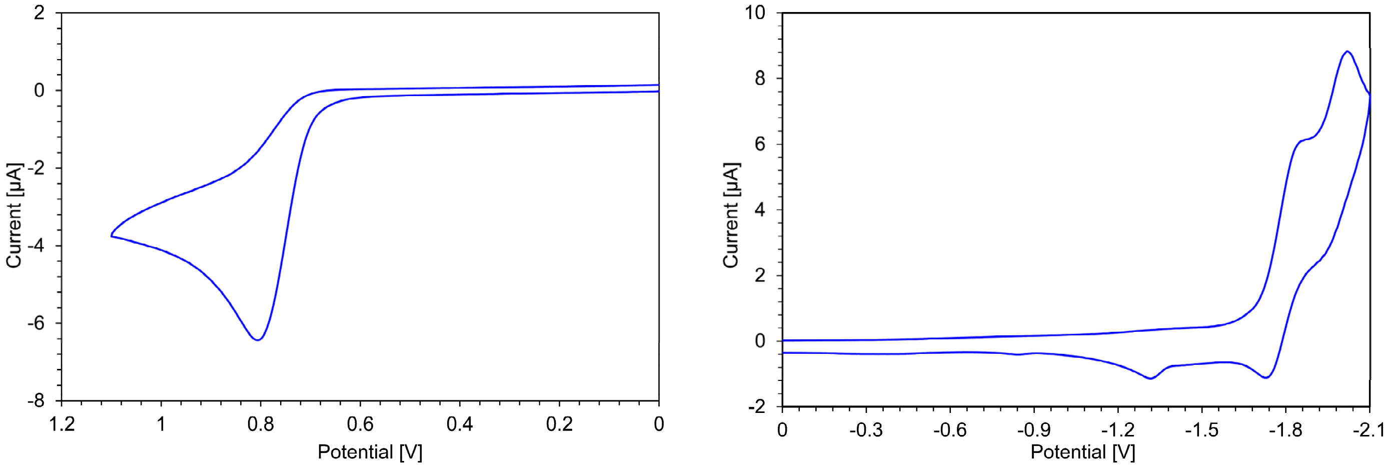


**Figure S24.** Cyclic voltammogram for oxidation (left) and reduction (right) of **7** (1 mM) in benzonitrile containing $Et_4NClO_4$ (0.1 M) as the supporting electrolyte; scan rate = 100 $mVs^{-1}$.

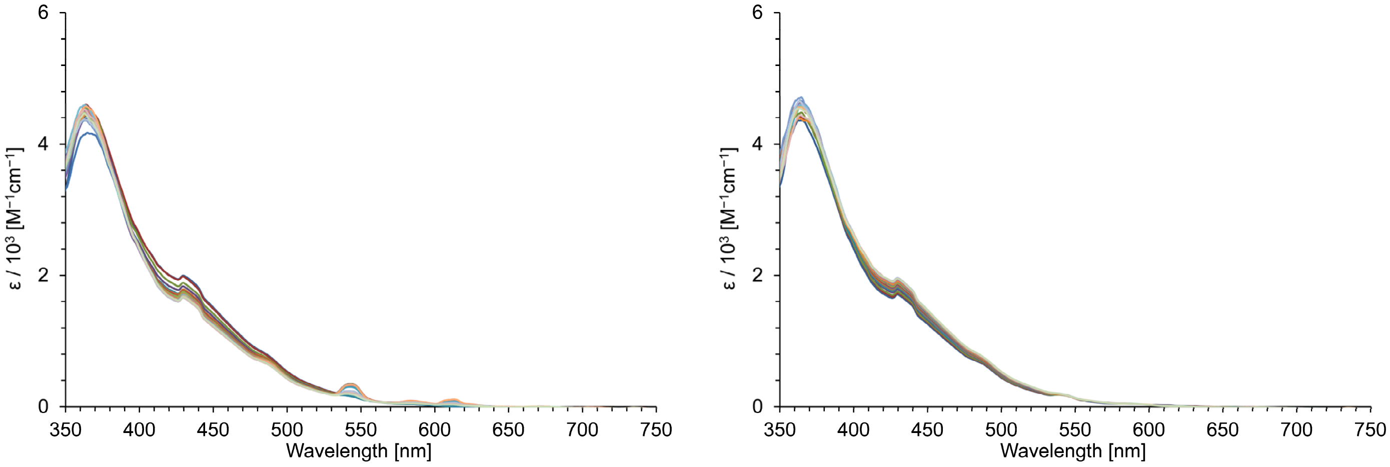


**Figure S25.** Continuous change in the visible spectrum of **7**: constant-voltage electrochemical oxidation at +1.10 V (left) and electrochemical reduction of the oxidized species at ±0 V (right) in benzonitrile containing $Et_4NClO_4$ (0.1 M) at 30 sec intervals.

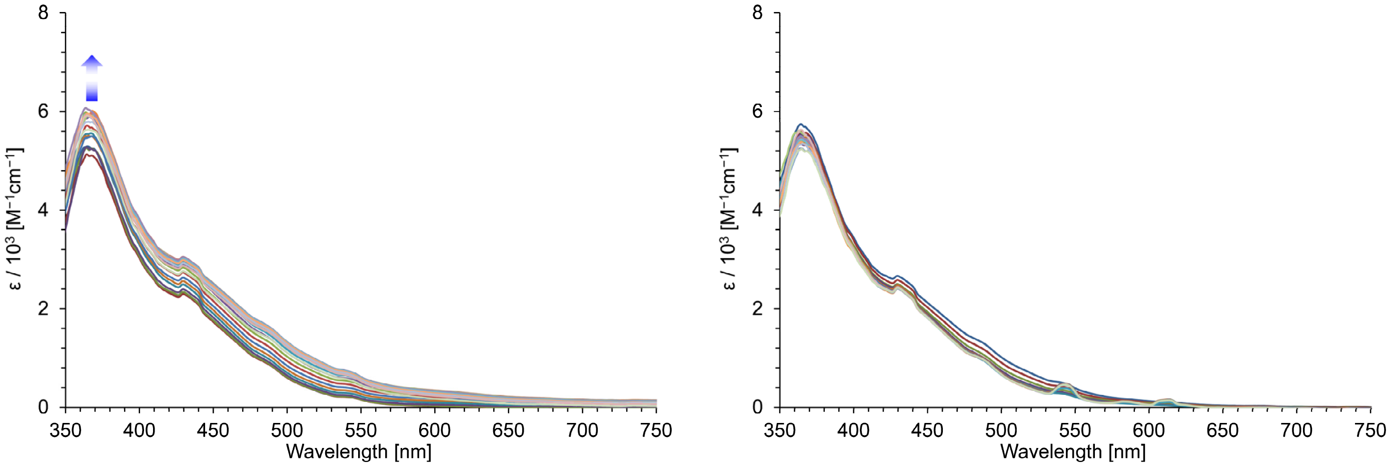


**Figure S26.** Continuous change in the visible spectrum of **7**: constant-voltage electrochemical reduction at −2.10 V (left) and electrochemical oxidation of the reduced species at ±0 V (right) in benzonitrile containing $Et_4NClO_4$ (0.1 M) at 30 sec intervals.

## 4. ORTEP Drawing of 6a,b and 7 (Figures S27–29).

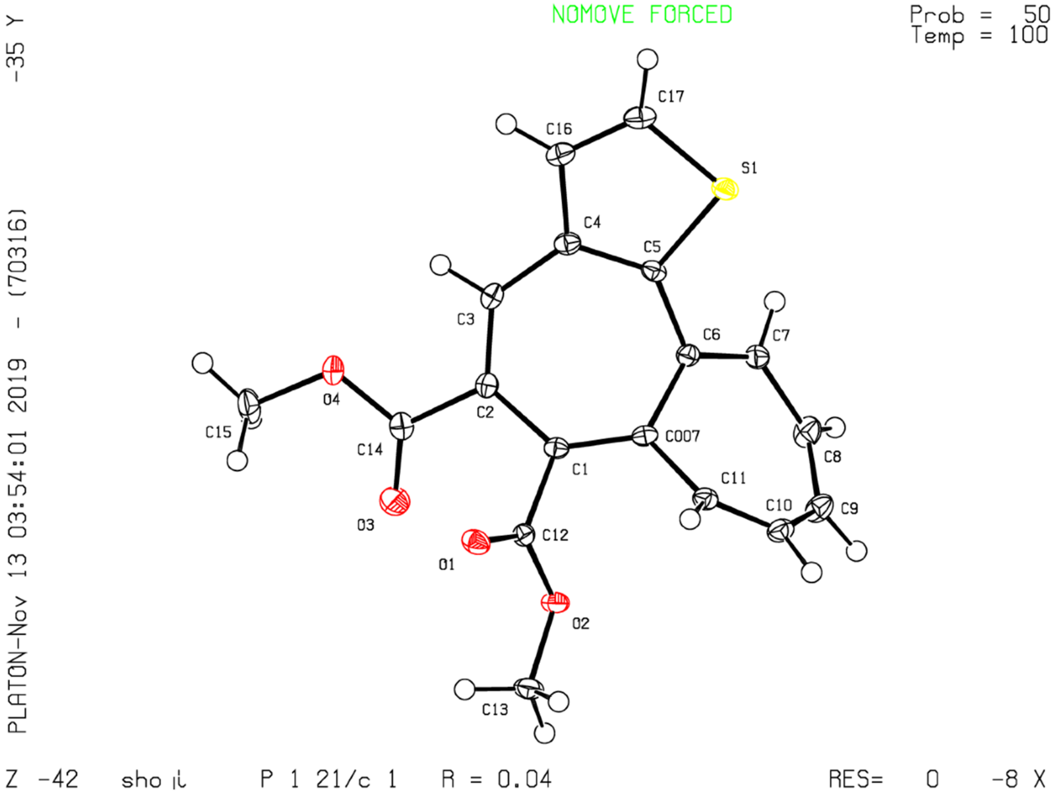


**Figure S27.** ORTEP diagrams of **6a** (**CCDC1965949**); ellipsoids are drawn at the 50% probability level.

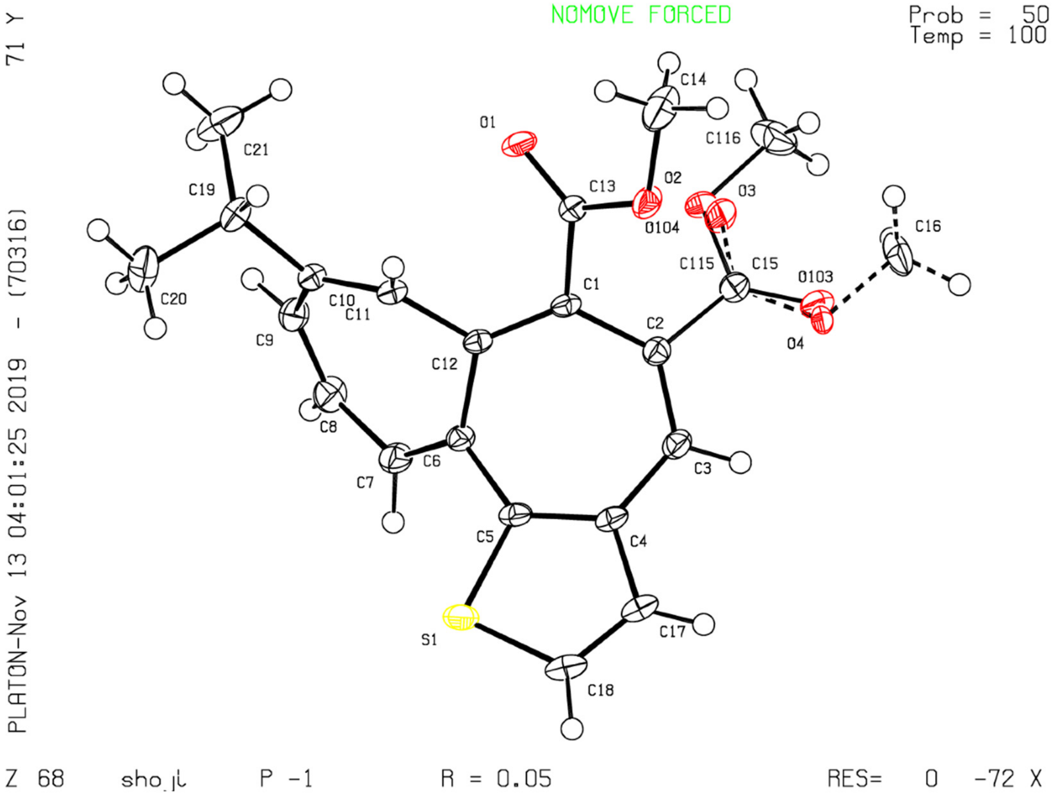


**Figure S28.** ORTEP diagrams of **6b** (**CCDC1965950**); ellipsoids are drawn at the 50% probability level.

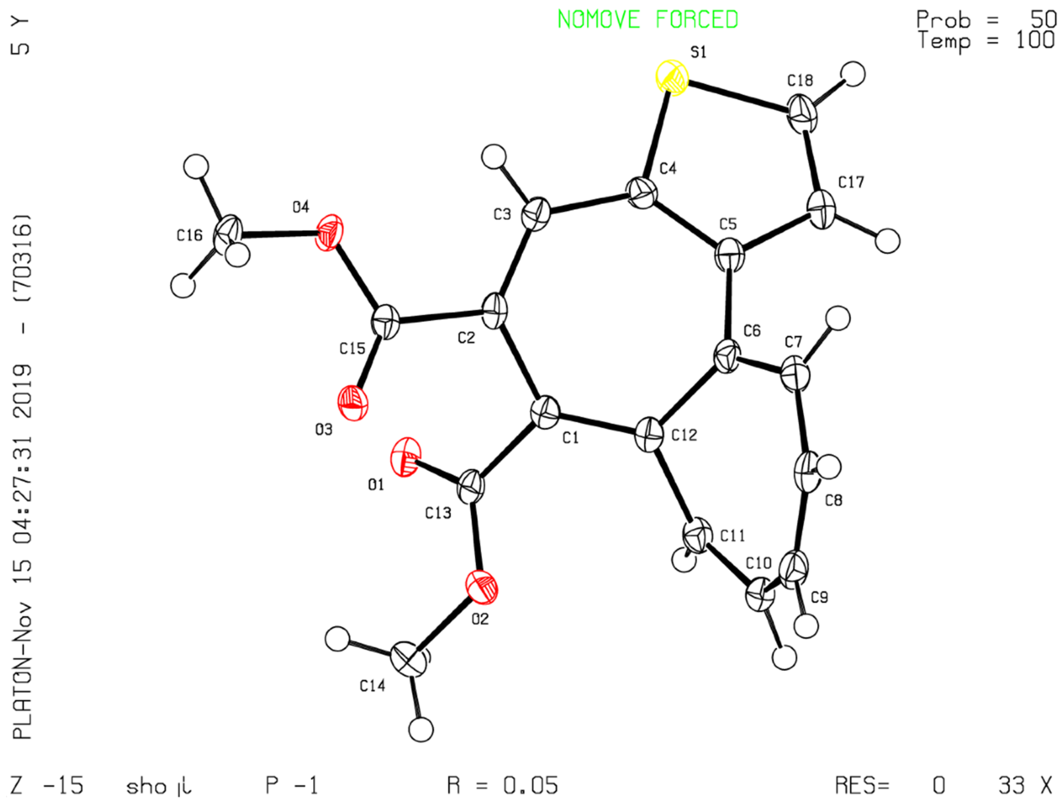


**Figure S29.** ORTEP diagrams of **7 (CCDC1965951)**; ellipsoids are drawn at the 50% probability level.
